# Exploring the interaction between endornavirus and *Sclerotinia sclerotiorum*: mechanisms of phytopathogenic fungal virulence and antivirus

**DOI:** 10.1128/mbio.03365-24

**Published:** 2025-02-19

**Authors:** Fan Mu, Jinsheng Xia, Jichun Jia, Daohong Jiang, Baojun Zhang, Yanping Fu, Jiaseng Cheng, Jiatao Xie

**Affiliations:** 1 College of Plant Protection, Shanxi Key Laboratory of Integrated Pest Managementin Agriculture, Shanxi Agricultural University, Jinzhong, Shanxi, China; 2 National Key Laboratory of Agricultural Microbiology, The Provincial Key Lab of Plant Pathology of Hubei Province, College of Plant Science and Technology, Huazhong Agricultural University, Wuhan, Hubei, China; 3 Hubei Hongshan Laboratory, Wuhan, Hubei, China; Cornell University, Ithaca, New York, USA

**Keywords:** *Sclerotinia sclerotiorum*, mycovirus, endornavirus, hypovirulence, RNAi, antivirus response

## Abstract

Hypovirulence-associated mycoviruses have the potential as biocontrol agents for plant fungal disease management, and exploration of the interactions between these mycoviruses and phytopathogenic fungi can provide opportunities to elucidate the underlying mechanisms of hypovirulence and antiviruses. We previously found that Sclerotinia sclerotiorum endornavirus 3 (SsEV3), belonging to the genus *Betaendornavirus* within the family *Endornaviridae*, confers hypovirulence on the phytopathogenic fungus *Sclerotinia sclerotiorum*, but the underlying mechanisms remains unclear. In this study, we found that the SsEV3-infected strain produced fewer sclerotia, failed to form infection cushions on plant hosts, exhibited increased cell vacuolation, and was more sensitive to abiotic stresses. SsEV3 infection evoked transcriptional rewiring in *S. sclerotiorum*, affecting genes related to virulence factors for pathogenicity and RNAi pathway for antiviruses. An unknown biological function of gene *Sssnf1* was downregulated following SsEV3 infection. Deletion of *Sssnf1* impaired infection cushion formation and decreased virulence of *S. sclerotiorum*. Five key RNAi-related genes were significantly upregulated, and deletion of *Ssdcl2* contributed to SsEV3 accumulation. Additionally, we identified a hypothetical protein encoded by *Sshp1* that directly interacts with the RNA-dependent RNA polymerase (RdRp) domain encoded by SsEV3. Although the deletion mutants of *Sshp1* exhibited normal colony morphology, they showed higher SsEV3 accumulation and reduced resistance to reactive oxygen species, indicating that this gene*,* similar to RNAi-related genes, plays an antiviral role in response to SsEV3 infection and may represent a new antivirus factor. Therefore, examination of the interaction between endornavirus and *S. sclerotiorum* provides new insights into the mechanisms of antivirus and virulence in phytopathogenic fungi.

**IMPORTANCE:**

Hypovirulence-associated mycoviruses have emerged as promising biocontrol agents, and studying their interactions with phytopathogenic fungi helps uncover mechanisms of fungal pathogenesis and antiviral defense. This study provides critical insights into the interaction between *Sclerotinia sclerotiorum* and its hypovirulence-associated endornavirus, SsEV3, elucidating the molecular mechanisms underlying mycovirus-induced changes in fungal virulence and antivirus defense. SsEV3 infection not only impairs fungal virulence traits, including infection cushion formation and sclerotial production but also triggers host antiviral responses involving typical RNA interference pathways. New virulence factors, such as *Sssnf1*, and antiviral factors, such as *Sshp1*, were identified based on the established interaction system between *S. sclerotiorum* and endornavirus. These findings deepen our understanding of fungus-mycovirus interactions, highlighting the role of SsEV3 in reducing the virulence of *S. sclerotiorum*, and facilitating the development of mycovirus-based biological control strategies.

## INTRODUCTION

Mycoviruses are ubiquitous across all major fungal taxa, with hypovirulence-associated mycoviruses offering the potential to control crop fungal diseases (1, 2). Investigating the interaction system between hypovirulence-associated mycoviruses and their hosts can elucidate the signaling pathways involved in fungal pathogenicity and antiviral defense mechanisms (3, 4). The interaction system of Cryphonectria parasitica hypovirus 1 (CHV1) and *Cryphonectria parasitica* has been studied at the molecular and cellular levels, focusing on the genes involved in symptom development, defense mechanisms, and viral transmission (3, 5). Additionally, interactions involving Fusarium graminearum hypovirus 1 in *Fusarium graminearum* (6, 7), Talaromyces marneffei partitivirus-1 in *Talaromyces marneffei* (8), Malassezia sympodialis mycovirus in *Malassezia sympodialis* (9), and Colletotrichum alienum partitivirus 1 in *Colletotrichum alienum* (10), have been investigated through RNA-seq analysis. Fungi employ antiviral defense mechanisms, including RNA silencing, to combat viral infections (11).

*Sclerotinia sclerotiorum*, a ubiquitous necrotrophic plant pathogen, can infect over 700 plant species, including important crops and weeds (12, 13). The virulence factors for *S. sclerotiorum* are primarily focused on cell wall-degrading enzymes, secreted proteins, and acidic substances (14–16). Additionally, infection cushions are crucial for the virulence of *S. sclerotiorum*. To date, 31 genes have been reported to regulate the formation of infection cushions, with gene knockout resulting in fewer infection cushions and decreasing virulence of *S. sclerotiorum* (14, 16). Several hypovirulence-associated mycoviruses with RNA or DNA genomes have been discovered in *S. sclerotiorum*, and their interaction systems have been established to reveal the molecular mechanisms underlying biological processes. For instance, the Sclerotinia sclerotiorum debilitation-associated RNA virus confers hypovirulence in strain Ep-1PN, leading to the downregulation of 150 genes, including *S. sclerotiorum* integrin-like gene (*SSITL*) (17). Targeted silencing of *SSITL* significantly reduces the virulence and growth rate of *S. sclerotiorum* (18). Sclerotinia sclerotiorum hypovirus 2-L (SsHV2-L) infection alters the genes involved in carbohydrate and lipid metabolism and trafficking, influencing the accumulation of mRNA and small RNA in *S. sclerotiorum* (19). Furthermore, Sclerotinia sclerotiorum hypovirulence-associated DNA virus 1 (SsHADV1) affects DNA replication, DNA damage response, ribosomal assembly, and translation of *S. sclerotiorum* (20–22).

Endornaviruses are positive single-stranded RNA viruses with genomes ranging from 9.7 to 17.6 kb, and they have been reported to infect plants, fungi, and oomycetes (23). The family *Endornaviridae* includes two genera, *Alphaendornavirus* and *Betaendornavirus*, with viruses classified based on genome size, host, and the presence of unique domains. A nick structure has been identified in *Alphaendornavirus*, but this structure has not yet been observed in *Betaendornavirus* (23). Most members of the family *Endornaviridae* exert no notable influence on their host phenotypes. Four endornaviruses, Helicobasidium mompa endornavirus 1 (24), Sclerotinia minor endornavirus 1 (25), Sclerotinia sclerotiorum endornavirus 3 (26), and Sclerotinia sclerotiorum endornavirus 11 (27), are associated with host hypovirulence. Despite these findings, there is still a limited understanding of the molecular mechanisms underlying these hypovirulence effects, and no interaction system exists to elucidate the interaction between endornaviruses and their fungal hosts. Sclerotinia sclerotiorum endornavirus 3 (SsEV3) is a primary hypovirulence factor in *S. sclerotiorum* strain SX276 (26). In this study, to explore the interaction between SsEV3 and *S. sclerotiorum*, we analyzed the biological characteristics and transcriptional responses of *S. sclerotiorum* to SsEV3 infection. These results suggested that the interaction between SsEV3 and *S. sclerotiorum* is multifaceted, involving specific changes in the expression of genes associated with virulence factors, and antiviral responses. This study provides new insights into the mechanisms of interactions between endornaviruses and *S. sclerotiorum*.

## RESULTS

### SsEV3 induces hypovirulence in *S*. *sclerotiorum*

**Fig 1:**
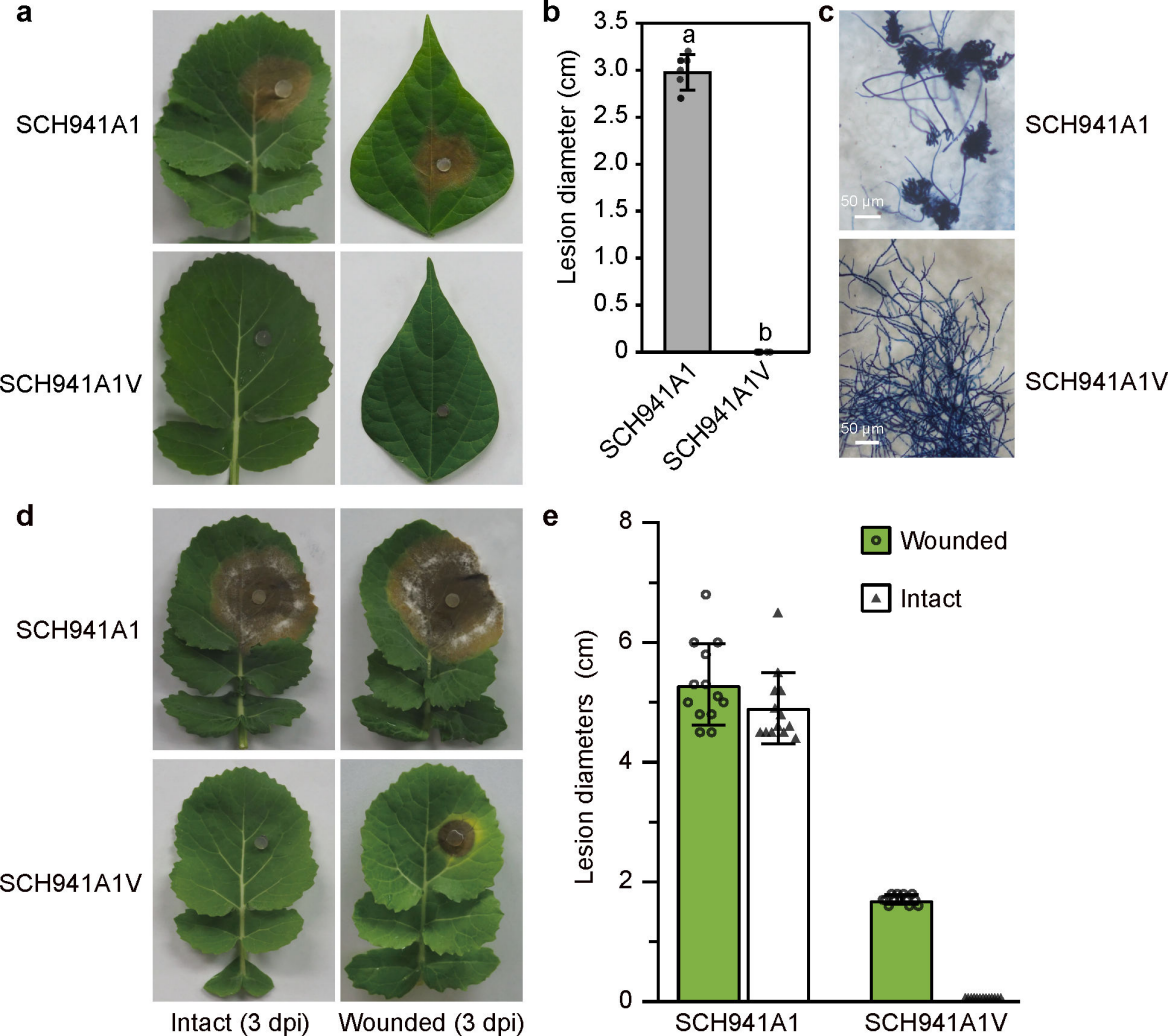
Biological characteristics of the SsEV3-infected strain SCH941A1V. (**a**) Virulence assay of *Sclerotinia sclerotiorum* virus-free strain SCH941A1 and SsEV3-infected strain SCH941A1V on detached rapeseed and soybean leaves at 48 hpi, 20°C. (**b**) Lesion diameters induced by the two strains on detached rapeseed leaves (20°C, 48 hpi). (**c**) Formation of infection cushions by strains SCH941A1 and SCH941A1V on rapeseed leaves. (**d**) Pathogenicity of strains SCH941A1 and SCH941A1V on detached intact rapeseed leaves and wounded rapeseed leaves at 3 dpi, 20°C. (**e**) Lesion diameters induced by the two strains on detached intact rapeseed leaves and wounded rapeseed leaves (20°C, 3 dpi).

We compared the virulence, sclerotial formation, infection cushions formation, and cellular structure of the SsEV3-infected strain SCH941A1V with the virus-free strain SCH941A1. Strain SCH941A1V exhibited significantly reduced virulence and failed to induce typical lesions on detached rapeseed or soybean leaves (Fig. 1a and b). Moreover, it did not form infection cushions on rapeseed leaves even at 36 hours post-inoculated (hpi), whereas stain SCH941A1 produced infection cushions at 12 hpi (Fig. 1c). However, strain SCH941A1V could cause lesions on wounded leaves (Fig. 1d and e), indicating that its failure to form lesions on detached leaves was likely because of its defective ability to form infection cushions. Both strains produced acidic substances, as evidenced by the color change in the pH-indicator medium at 3 days post-inoculation (dpi) (Fig. S1). However, strain SCH941A1V failed to degrade the acidic substances at the later growth stages, as the pH-indicating medium remained yellow at 10 dpi, whereas the growth of strain SCH941A1 caused the medium to shift from yellow to blue (Fig. S1).

**Fig 2:**
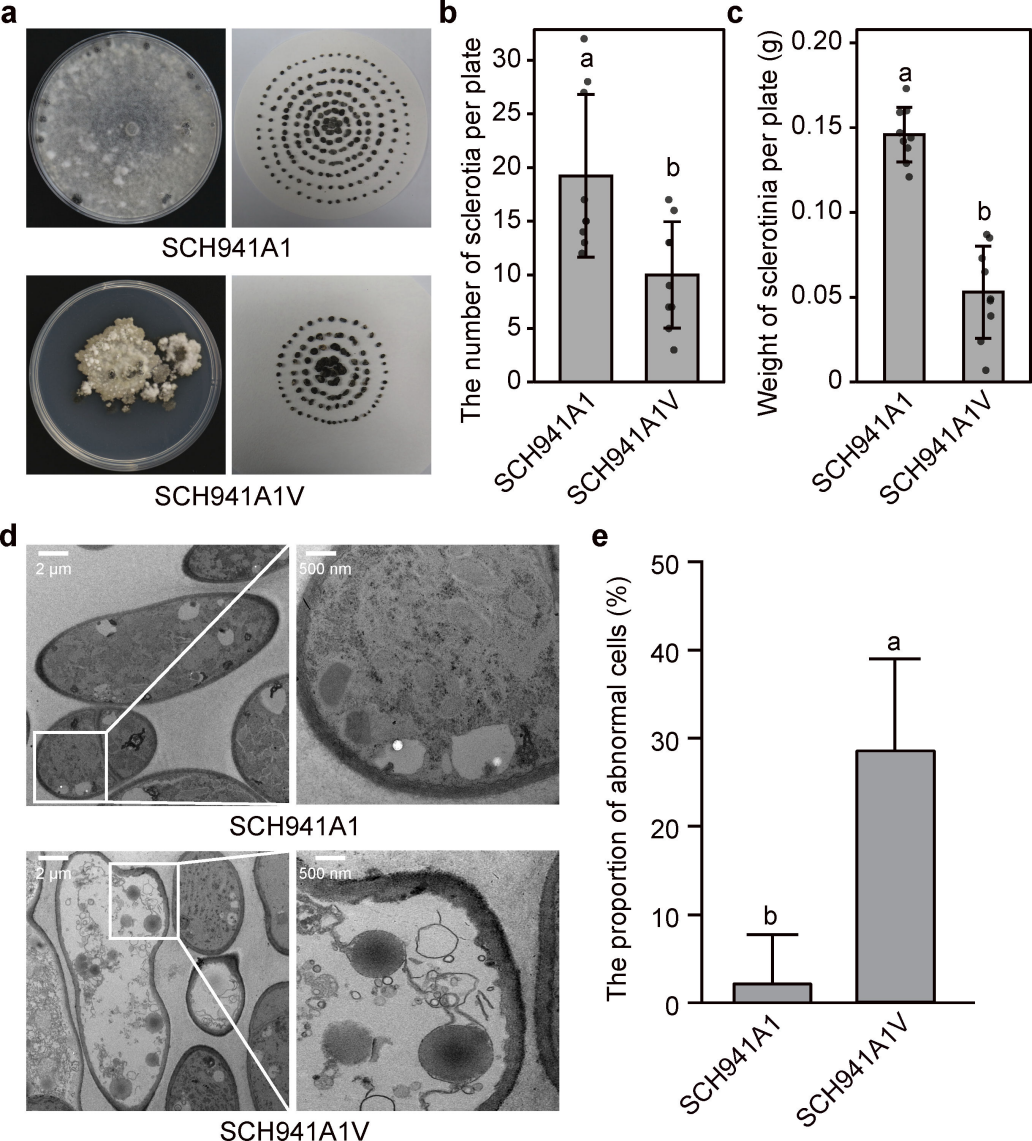
Sclerotial and cellular ultrastructure of virus-free and SsEV3-infected strains. (**a**) Sclerotia of strains SCH941A1 and SCH941A1V. Sclerotia from 10 PDA plates of each strain were collected and photographed together. (**b**) Average sclerotial number of strains SCH941A1 and SCH941A1V obtained from 10 PDA plates. (**c**) Average sclerotial weight of strains SCH941A1 and SCH941A1V obtained from 10 plates. (**d**) Cellular ultrastructure of virus-free and SsEV3-infected strains. Transmission electron microscopy images of the hyphae of strains SCH941A1 and SCH941A1V were taken after being cultured for 2 days on PDA. (**e**) The average proportion of abnormal cells in strains SCH941A1 and SCH941A1V. The total number of cells in the five visual fields and the number of abnormal cells were counted, and the proportion of abnormal cells was calculated.

Both SCH941A1 and SCH941A1V produced sclerotia on potato dextrose agar (PDA) at 7 dpi (Fig. 2a). However, strain SCH941A1V exhibited impaired sclerotial formation compared to SCH941A1. The number and weight of sclerotia produced by strain SCH941A1V were reduced by 50% and 63%, respectively, compared with those produced by strain SCH941A1 (Fig. 2b and c).

The effect of SsEV3 on the cellular structure of *S. sclerotiorum* was assessed. In strain SCH941A1, the hyphal cells remained intact, exhibiting normal cell wall integrity and well-organized cellular components (Fig. 2d). In contrast, SCH941A1V cells showed damaged and blurred cell membranes, indistinct and incomplete organelles, and an increased number of vacuoles (Fig. 2d). Statistical analyses revealed that vacuolated cells constituted 28.8% of SCH941A1V cells, compared to only 2.5% in SCH941A1 cells (Fig. 2e; Fig. S2; Table S1). These results indicated that SsEV3 significantly affected the cellular structure of *S. sclerotiorum*.

### SsEV3 infection increases sensitivity to abiotic stresses

**Fig 3:**
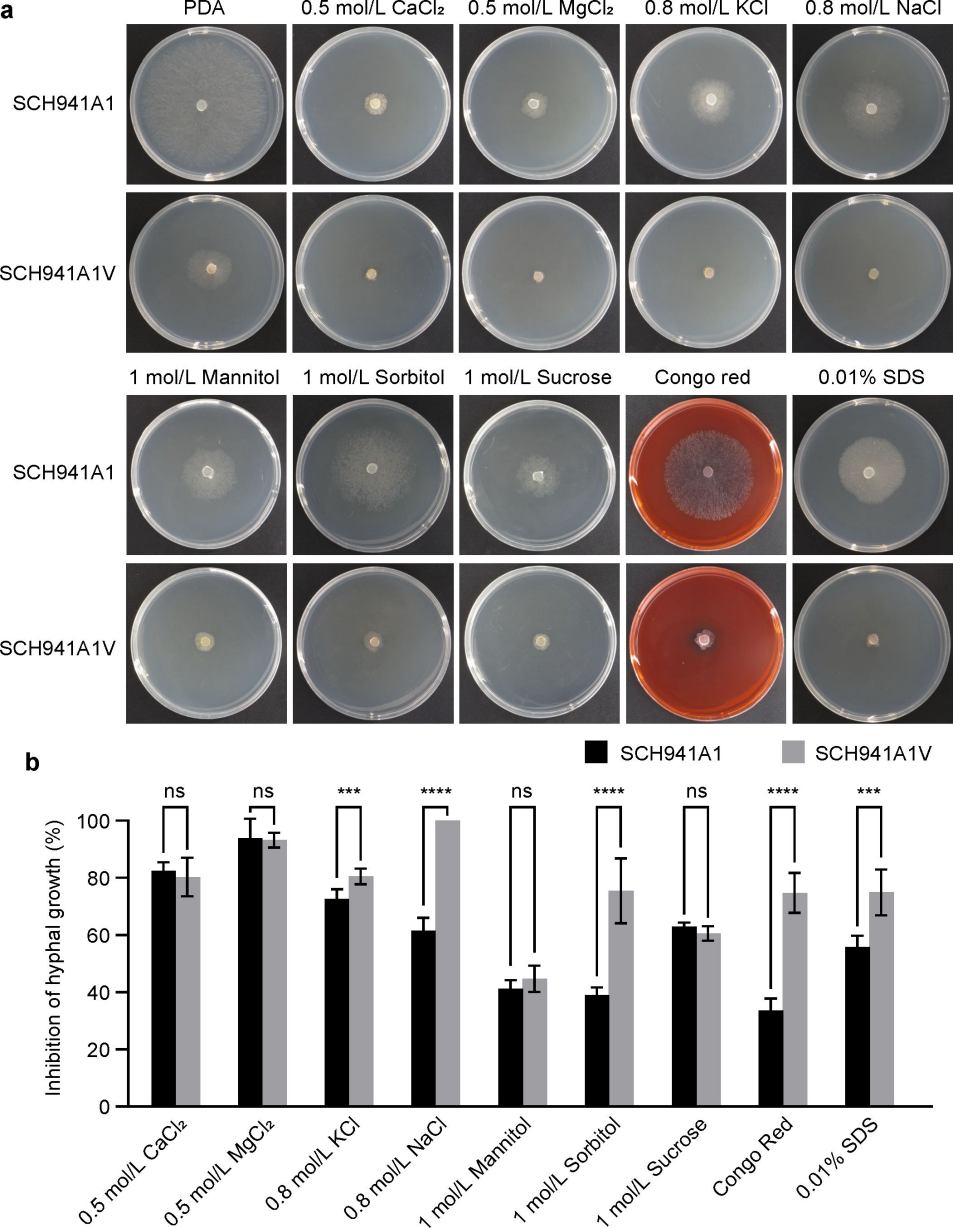
Growth assay of SsEV3-infected strain SCH941A1V under different abiotic stresses. (**a**) Colony morphology of virus-free and SsEV3-infected strains on various abiotic stresses. The photographs were taken after 2 days of growth at 20°C. (**b**) Relative inhibition of *S. sclerotiorum* hyphal growth in response to different abiotic stresses.

Considering the critical role of the fungal cell walls in maintaining morphology and protecting against environmental stresses (28), we further investigated whether the alterations in cellular structure affected the sensitivity of *S. sclerotiorum* to abiotic stresses. Colony morphology and growth rates of strains SCH941A1V and SCH941A1 were assessed in response to different abiotic stresses, including osmotic and cell wall stressors. High concentrations of KCl (0.8 M), NaCl (0.8 M), sorbitol (1 M), and cell wall stressors (Congo Red and sodium dodecyl sulfate [SDS]) inhibited hyphal growth in both strains, however, strain SCH941A1V was more sensitive to these stressors (Fig. 3a). The growth inhibition rate of abiotic stresses on strain SCH941A1 ranged from 38.9% to 93.8%, whereas in strain SCH941A1V these ranged from 44.7% to 100% (Fig. 3b).

### SsEV3 evokes transcriptional rewiring in *S. sclerotiorum*

**Fig 4:**
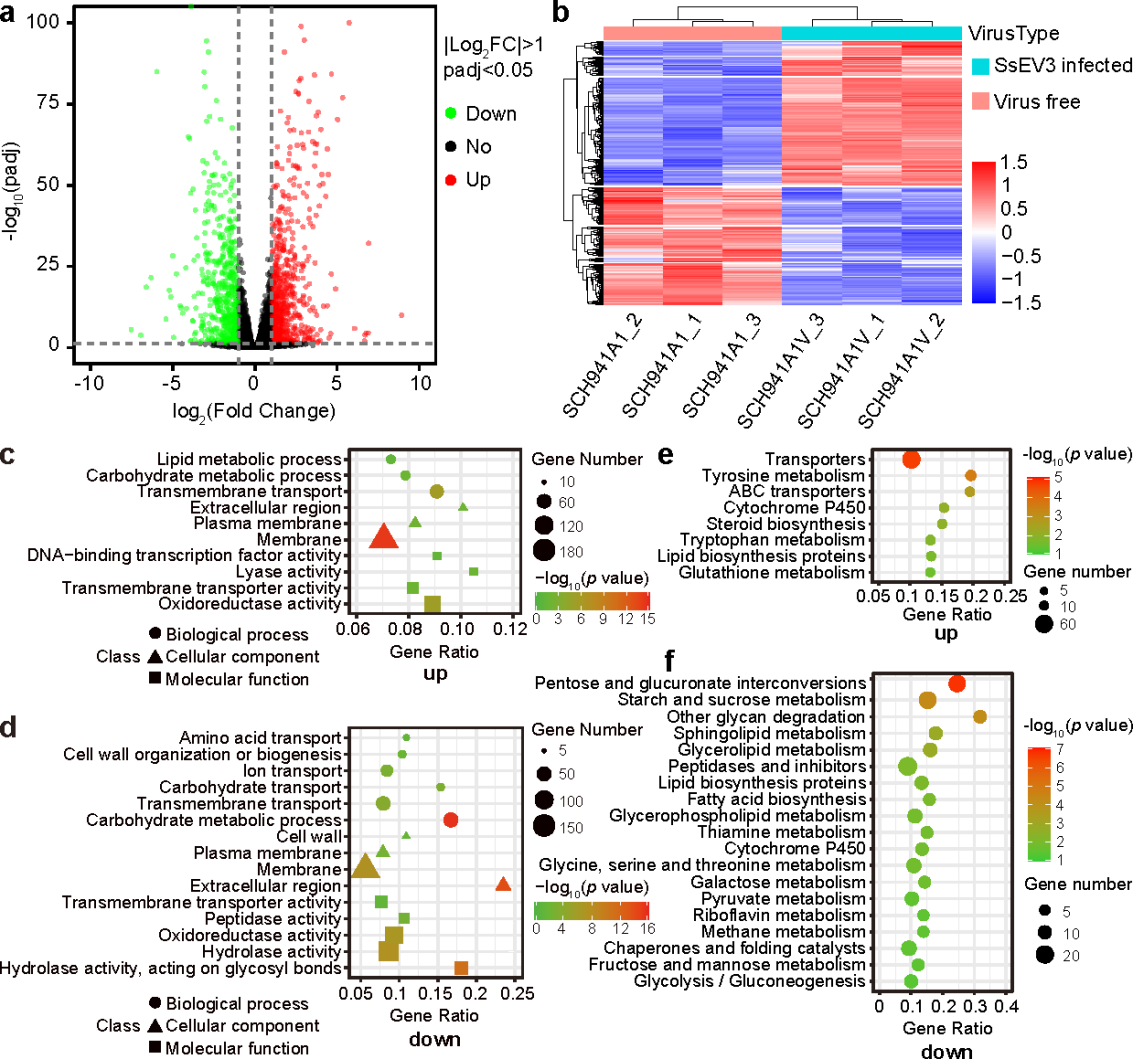
Transcriptome analysis of DEGs upon SsEV3 infection. (**a**) Volcano plot of the genes in the virus-free and SsEV3-infected strains. The green dots represent the downregulated genes; the red dots represent the upregulated genes; and the black dots represent genes not expressed differentially. (**b**) Heatmap of DEGs between virus-free and SsEV3-infected strains. (**c**) GO enrichment analysis of the upregulated genes. (**d**) GO enrichment analysis of the downregulated genes. (**e**) Kyoto Encyclopedia of Genes and Genomes (KEGG) pathway enrichment analysis of upregulated genes. (**f**) KEGG pathway enrichment analysis of the downregulated genes.

To investigate the transcriptional responses of *S. sclerotiorum* to SsEV3 infection, we conducted RNA-seq analysis during vegetative growth of strains SCH941A1 and SCH941A1V. Over 6 GB of data were obtained from each sample, with average alignment rates of 95.2% and 96.1% to the *S. sclerotiorum* genome, respectively (Table S2). Both principal component analysis (PCA) and Pearson correlation coefficient analyses showed strong correlations among the sequenced replicate samples, ensuring the repeatability of the sequenced data for further analysis (Fig. S3). Differential gene expression analysis identified 1,339 differentially expressed genes (DEGs) upon SsEV3 infection, with 594 upregulated and 745 downregulated genes (Fig. 4a and b).

The DEGs were mapped to the Gene Ontology (GO) databases to determine their potential functions. GO terms analysis revealed that the upregulated genes were significantly enriched in categories “Lipid metabolic process,” “Carbohydrate metabolic process,” “Transmembrane transport,” “Extracellular region,” “Plasma Membrane,” “Membrane,” “DNA-binding transcription factor activity,” “Lyase activity,” “Transmembrane transporter activity,” and “Oxidoreductase activity” (Fig. 4c). Fifteen GO terms were significantly enriched among the 745 downregulated genes, with multiple GO terms related to material transport, including “Amino acid transport,” “Ion transport,” “Transmembrane transport,” and “Transmembrane transporter activity.” Additionally, “Cell wall,” “Plasma membrane,” and “Membrane” were also enriched (Fig. 4d). These results suggest that SsEV3 infection may impair the substance transport in *S. sclerotiorum* and affect cell structure, potentially explaining the slow growth of strain SCH941A1V on PDA and its inhibition under abiotic stress conditions.

To identify the pathways significantly regulated by SsEV3 infection, the DEGs were mapped to the Kyoto Encyclopedia of Genes and Genomes (KEGG) database. Eight KEGG pathways were significantly enriched among the 594 upregulated genes (Fig. 4e). The top five enriched pathways were “Transporters,” “Tyrosine metabolism,” “ABC transporters,” “Cytochrome P450,” and “Steroid biosynthesis.” The enrichment of the “Transporters” and “ABC transporters” pathways was consistent with the GO term results, suggesting that SsEV3 infection might enhance carbohydrate acquisition in strain SCH941A1V. Nineteen KEGG pathways were significantly enriched among the 745 downregulated genes. Pathways related to energy metabolism, including “Pentose and glucuronate interconversions,” “Starch and sucrose metabolism,” and “Glycolysis/Gluconeogenesis” were significantly downregulated (Fig. 4f). These results demonstrated that SsEV3 infection may affect energy metabolism in *S. sclerotiorum*.

### SsEV3 downregulates genes related to virulence factors of *S. sclerotiorum*

**Fig 5:**
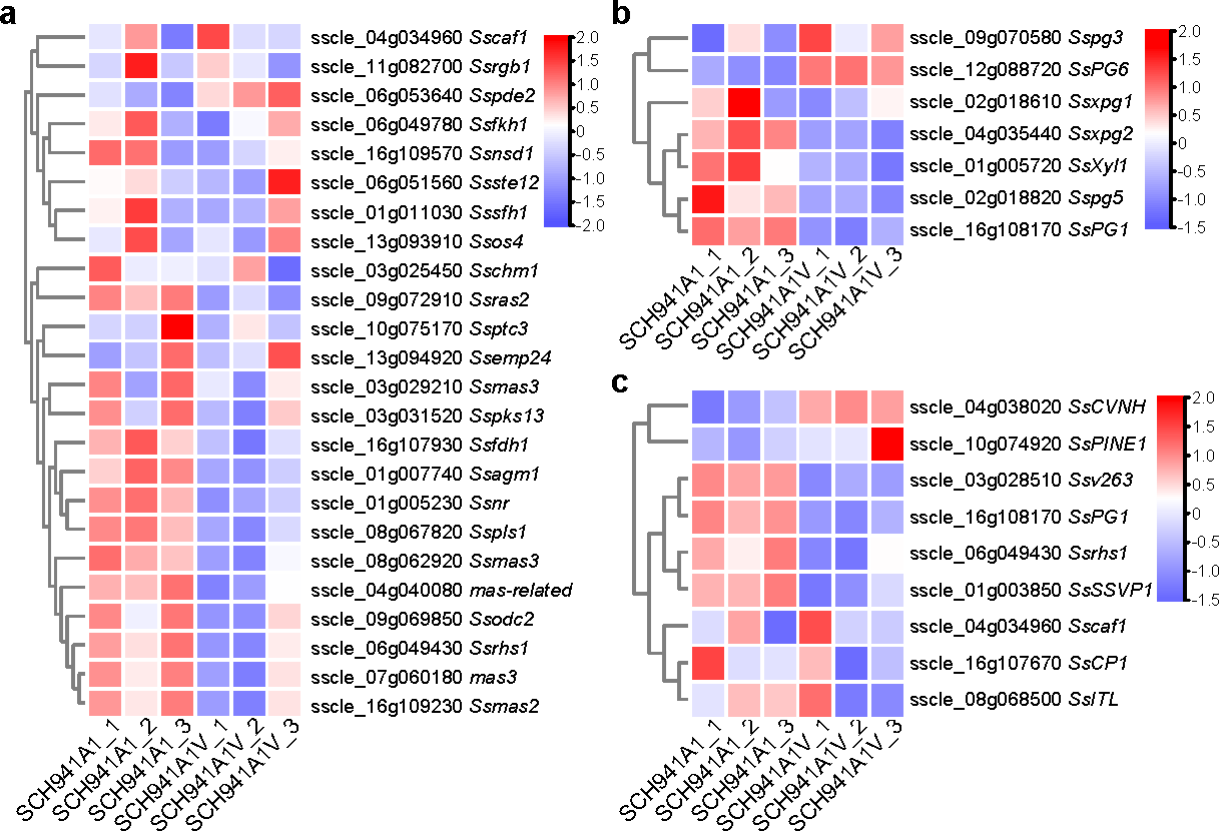
Expression of virulence factor-related genes in strains SCH941A1 and SCH941A1V. (**a**) Expression of infection cushions associated genes. (**b**) Expression of the reported cell wall-degrading enzymes-related genes. (**c**) Expression of the reported secretory protein genes.

A total of 44 homologous genes related to infection structures in *S. sclerotiorum* and *Magnaporthe oryzae* were identified (Fig. S4). Of these genes, 24 were downregulated following SsEV3 infection (Fig. 5a), potentially associated with abnormal infection cushion formation in strain SCH941A1V. For instance, *sscle_08*g062920 (*Ssmas3*), a homolog of *Momas3* related to virulence in *M. oryzae* (29), was downregulated by more than twofold in strain SCH941A1V. Among the seven previously identified genes encoding plant cell wall-degrading enzymes in *S. sclerotiorum* (14), five genes were downregulated in strain SCH941A1V, except for *sscle_09*g070580 (*SsPG3*) and *sscle_12*g088720 (*SsPG6*) (Fig. 5b). Furthermore, a total of 437 carbohydrate-active enZYmes (CAZymes) were predicted in genome of *S. sclerotiorum*, of which 126 were differentially expressed after SsEV3 infection, with downregulated genes accounting for 69% (87 downregulated and 39 upregulated genes) (Fig. S5a). Nine secretory proteins were previously reported to be related to the virulence in *S. sclerotiorum* (Fig. 5c). Apart from *SsCVNH*, eight secretory protein-related genes (*SsPINE1*, *Ssv263*, *SsPG1*, *SsRhs1*, *SsSSVP1*, *Sscaf1*, *SsCP1*, and *SSITL*) were downregulated in strain SCH941A1V (Fig. 5c). Additionally, the expression of the 486 genes related to secreted proteins identified in the *S. sclerotiorum* genome has been analyzed (30). In total, 126 genes were differentially expressed, with downregulated genes accounting for 76% (96 downregulated and 30 upregulated genes) (Fig. S5b). These results demonstrate that SsEV3 influences the pathogenicity of *S. sclerotiorum* by downregulating the expression of virulence factors, including genes related to infection cushion formation, cell wall-degrading enzymes, and secretory proteins.

**Fig 6:**
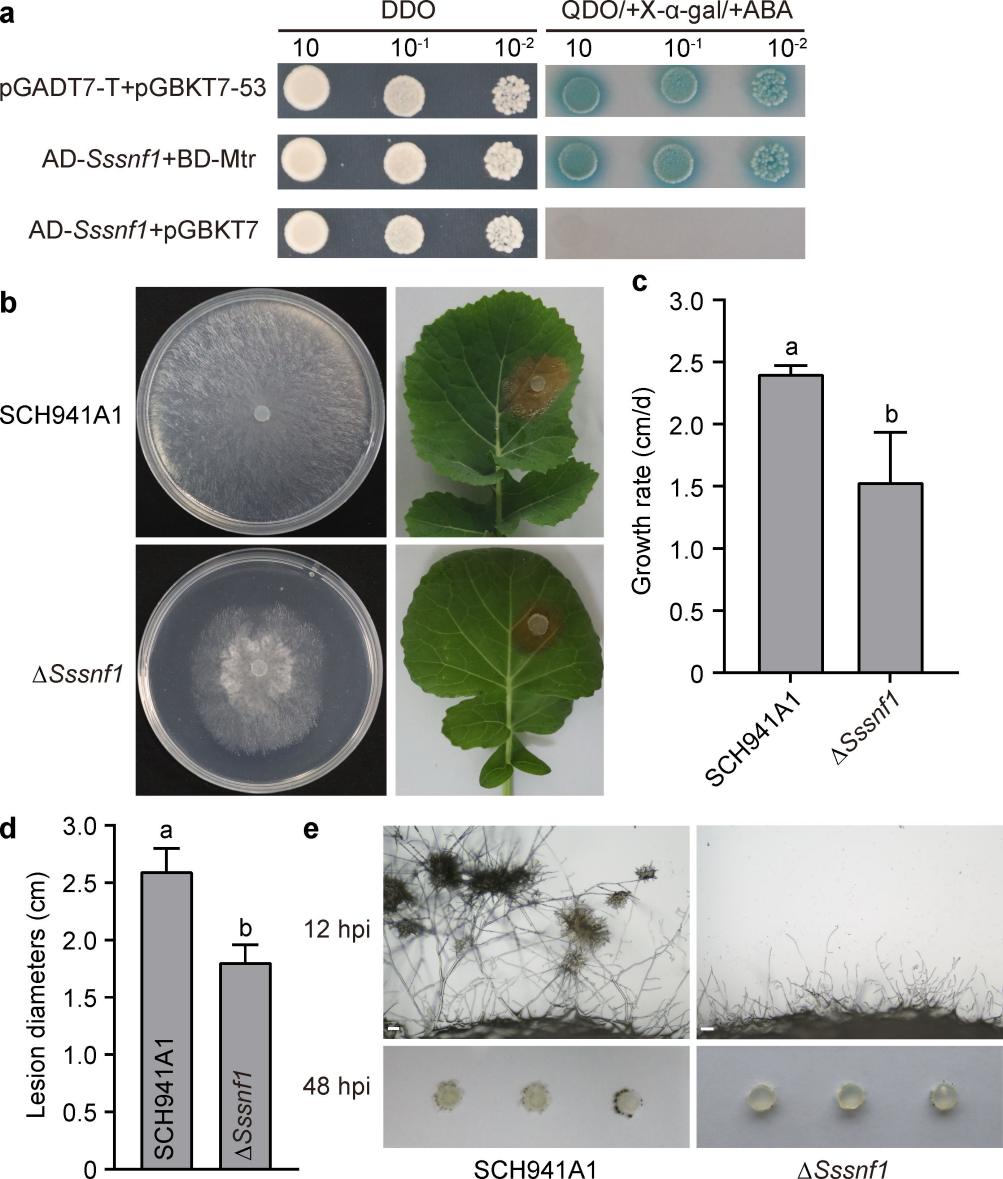
Biological characteristics of the ∆*Sssnf1* deletion mutant. (**a**) Yeast two-hybrid assay to determine the interaction between *Sssnf1* and the Mtr domain of SsEV3. pGADT7-T and pGBKT7-53 were used as positive controls, whereas AD-Sssnf1 and pGBKT7 were used as negative controls. DDO: SD/-Leu/-Trp medium, QDO/+X-α-gal/+ABA: SD/-Ade/-His/-Leu/-Trp medium containing 200 ng/mL ABA and 40 µg/mL X-α-gal. (**b**) Colony morphology and pathogenicity of *∆Sssnf1* deletion strain and SCH941A1. Colony morphology was photographed at 2 dpi on PDA. The pathogenicity on detached rapeseed leaves was evaluated and images were taken at 48 hpi. (**c**) Growth rate of strains ∆*Sssnf1* and SCH941A1 at 20°C. (**d**) Lesion diameters induced by the two strains on detached rapeseed leaves (20°C, 48 hpi). (**e**) Development of infection cushions. The mycelial plugs were inoculated on a glass slide and observed through an electron microscope at 12 hpi. The glass slide was photographed at 48 hpi.

To investigate the interaction between SsEV3 and *S. sclerotiorum*, a yeast two-hybrid assay was employed to identify *S. sclerotiorum* genes interacting with SsEV3. The results revealed that the protein encoded by *sscle_01*g011030 directly interacts with the methyltransferase (Mtr) domain of SsEV3 (Fig. 6a). Furthermore, this gene was found to be associated with the formation of infection cushions. The full-length sequence of the *sscle_01*g011030 gene is 1,841 bp and contains an open reading frame (ORF) with two exons. This ORF encodes a protein of 597 amino acids that harbors a conserved sucrose non-fermentable 5 (SNF5) motif (Fig. S6a). Based on these findings, the gene was designated as *Sssnf1*. The gene was slightly downregulated in the SsEV3-infected strain SCH941A1V. A deletion mutant was generated to explore its biological functions in *S. sclerotiorum* (Fig. S7a). The ∆*Sssnf1* deletion strain exhibited abnormal colony morphology with a significantly lower growth rate (Fig. 6b and c). Pathogenicity assays on detached rapeseed leaves showed that the lesions caused by the ∆*Sssnf1* deletion strain were markedly smaller compared to strain SCH941A1 (Fig. 6b and d). Additionally, infection cushion formation was significantly impaired in ∆*Sssnf1* deletion strain (Fig. 6e). Besides, to assess the effect of *Sssnf1* on SsEV3 accumulation, SsEV3 was horizontally transferred into ∆*Sssnf1* deletion mutant through mycelium fusion and confirmed by real-time reverse transcriptase PCR (RT-PCR) (Fig. S8a and b). Both deletion mutant and SsEV3-carrying deletion mutant ∆Sssnf1V strain could produce acid substances (Fig. S9), but compared to strain SCH941A1V, SsEV3-infected strain ∆Sssnf1V showed a slower growth rate and more deformed colony morphology (Fig. S8d through f). Quantitative real-time reverse transcriptase PCR (qRT-PCR) analysis showed that compared with SCH941A1V, SsEV3 accumulation in ∆Sssnf1V is 1.51 to 2.11 times higher (Fig. S8c), indicating enhanced SsEV3 replication in the absence of *Sssnf1* in *S. sclerotiorum*. Therefore, *Sssnf1* might play critical roles in vegetative growth, infection cushion formation, pathogenicity, and antivirus of *S. sclerotiorum*.

### SsEV3 infection triggers antivirus response involved in RNA silencing

**Fig 7:**
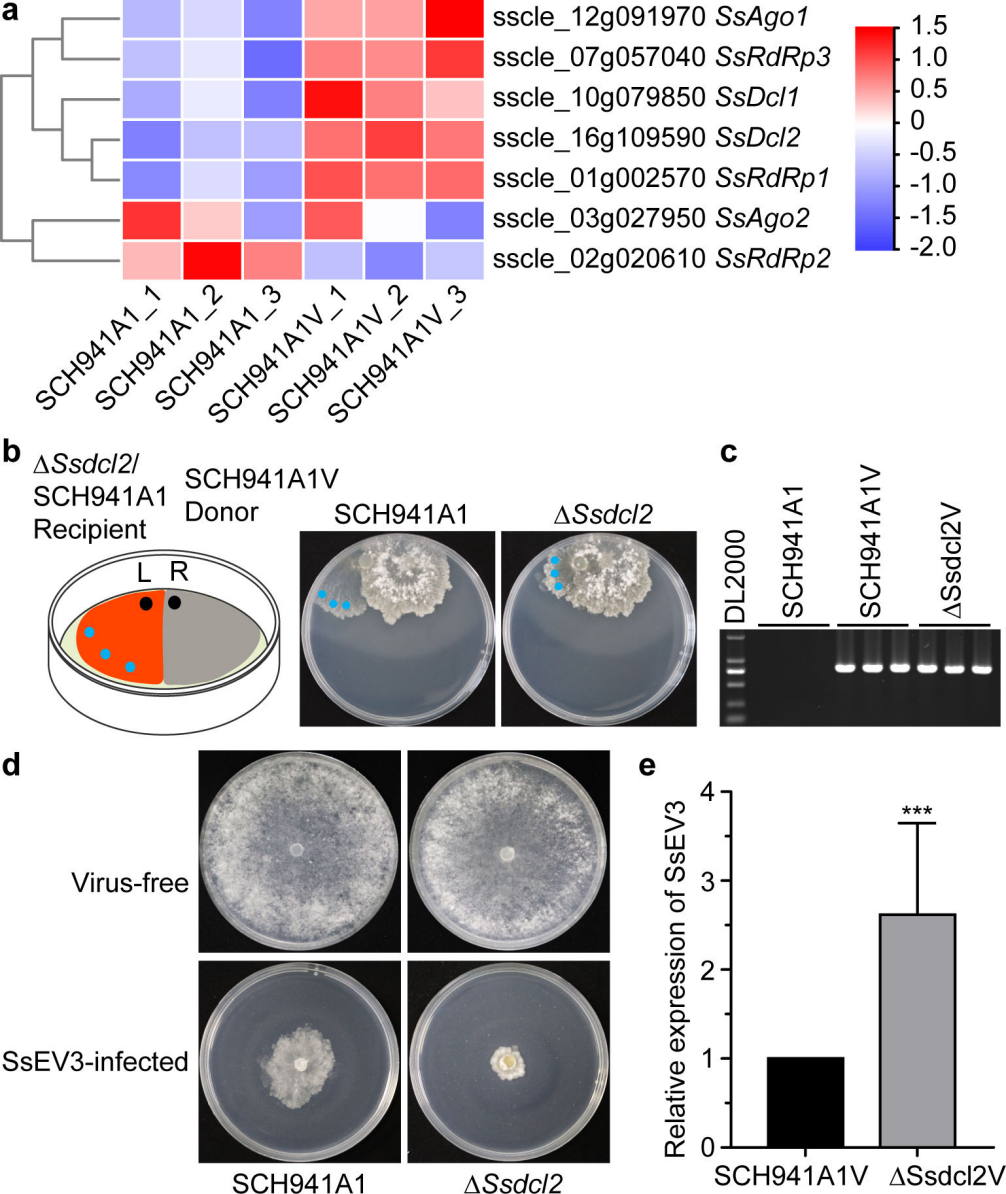
Expression of genes related to RNA silencing in *S. sclerotiorum* and the function of *Ssdcl2* upon SsEV3 infection. (**a**) Expression of RNA silencing associated genes in strains SCH941A1 and SCH941A1V. (**b**) Dual-culture of Δ*Ssdcl2* and SCH941A1V. Strain SCH941A1V on the right of plates served as the donor strain, Δ*Ssdcl2* was labeled with the hygromycin B resistance gene and served as the recipient strain, and SCH941A1 served as the recipient strain as control. The photos were taken for 3 days of co-culturing. (**c**) RT-PCR confirmation of SsEV3 in strain ΔSsdcl2V and SCH941A1V. (**d**) Colony morphology of Δ*Ssdcl2*, SCH941A1, ΔSsdcl2V, and SCH941A1V. The photos were taken after 3 days of incubation on PDA. (**e**) Relative expression of a gene from SsEV3 in strains ΔSsdcl2V and SCH941A1V using qRT-PCR.

Typical RNA-silencing-related genes (*Ssdcl1*, *Ssdcl2*, *SsRdRp1*, *SsRdRp3*, and *Ssago1*) in *S. sclerotiorum* were significantly upregulated following SsEV3 infection (Fig. 7a). To further explore the interaction between SsEV3 and RNAi-related genes, *Ssdcl2* was deleted in *S. sclerotiorum* through a split-marker approach and confirmed by serial PCR (Fig. S7b), and the resulting strain was named ∆*Ssdcl2* deletion. A SsEV3-infected strain, ∆Ssdcl2V, was created by dual-culturing of the ∆*Ssdcl2* deletion strain with the SsEV3-infected strain SCH941A1V (Fig. 7b). RT-PCR confirmed the successful infection of SsEV3 in ∆Ssdcl2V (Fig. 7c). The ∆*Ssdcl2* deletion strain showed the similar growth and morphology to strain SCH941A1, while SsEV3-infected strain ∆Ssdcl2V exhibited a significantly reduced growth rate compared to SCH941A1V (Fig. 7d). qRT-PCR analysis suggested that SsEV3 accumulation was 3.1-fold higher in ∆Ssdcl2V than in strain SCH941A1V (Fig. 7e), and siRNA derived from SsEV3 was significantly reduced in ∆Ssdcl2V (Fig. S7d) suggesting that *Ssdcl2* may play a direct antiviral role in response to SsEV3 infection.

### *S. sclerotiorum* protein directly interacts with SsEV3 to contribute to antiviral responses

**Fig 8:**
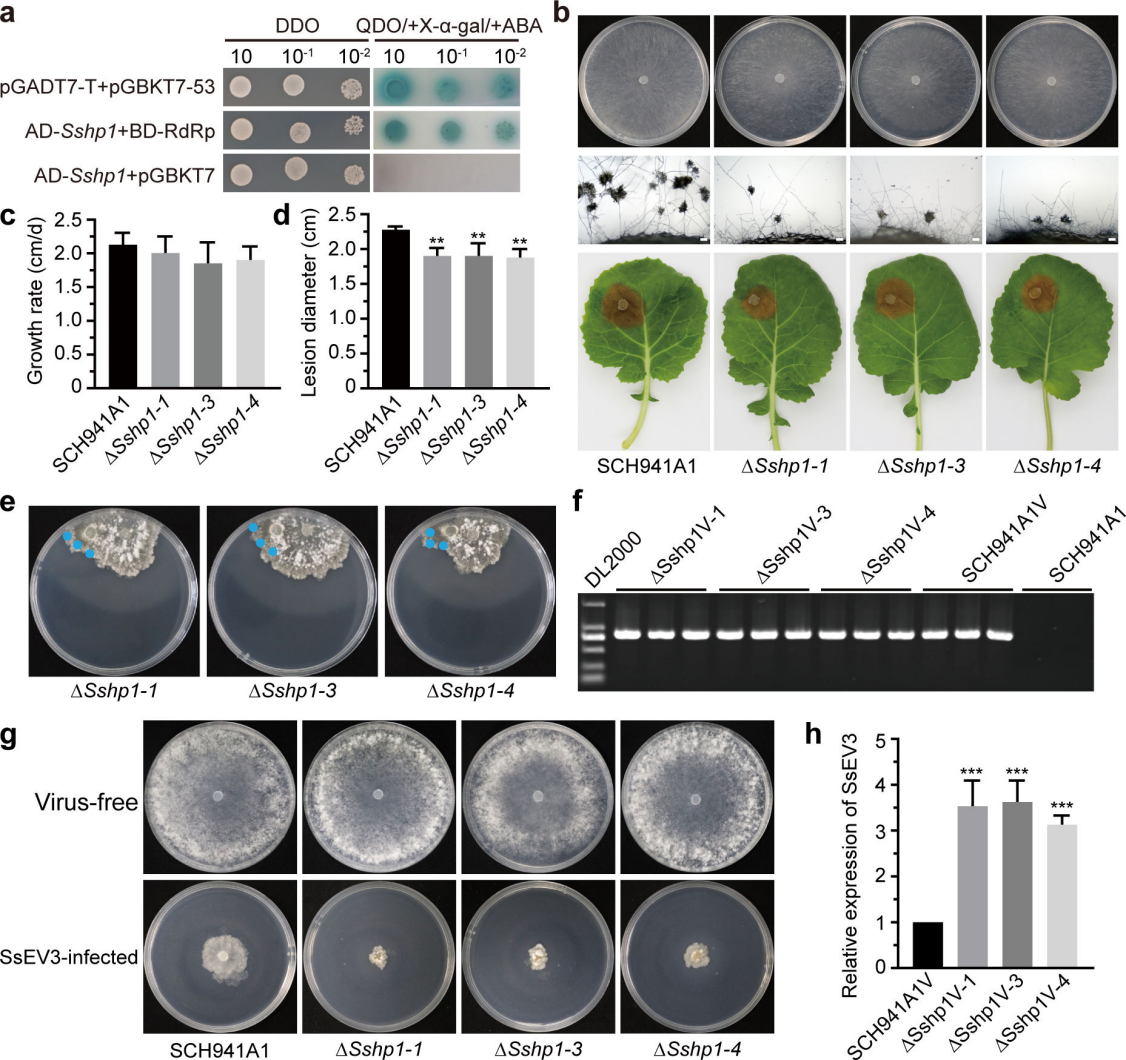
Biological characteristics of the ∆*Sshp1* deletion mutants. (**a**) Yeast two-hybrid assays were used to determine the interaction between *Sshp1* and the RdRp-domain of SsEV3. pGADT7-T and pGBKT7-53 were used as positive controls, while AD-Sshp1 and pGBKT7 were used as negative controls. DDO: SD/-Leu/-Trp medium, QDO/+X-α-gal/+ABA: SD/-Ade/-His/-Leu/-Trp medium containing 200 ng/mL ABA and 40 µg/mL X-α-gal. (**b**) Colony morphology, pathogenicity, and infection cushions of ∆*Sshp1* deletion mutants and SCH941A1. Photographs of colony morphology were taken after 3 days of incubation on PDA. The hyphal agar plugs were placed on glass slides, and the infection cushions were observed through an electron microscope at 12 hpi, the bar represents 50 µm. Photographs depicting pathogenicity and corresponding data were collected at 48 hpi on detached rapeseed leaves. (**c**) Growth rate of *∆Sshp1* deletion mutants and SCH941A1 at 20°C. (**d**) Lesion diameters induced by the deletion mutants and SCH941A1 on detached rapeseed leaves (20°C, 48 hpi). (**e**) Dual-cultured of ∆*Sshp1* deletion mutants and SCH941A1V on PDA. The photos were photographed after 3 days of co-culturing. (**f**) RT-PCR confirmation of the presence of SsEV3 in strain ∆Sshp1V and SCH941A1V. (**g**) Colony morphology of virus-free strains, and SsEV3-infected ∆Sshp1V and SCH941A1V. The photos were taken after 3 days of incubation on PDA. (**h**) Relative expression of a gene from SsEV3 in *∆Sshp1* deletion mutants infected by SsEV3 using qRT-PCR.

The yeast two-hybrid results demonstrated that the protein encoded by *sscle_03*g029490 directly interacts with the RNA-dependent RNA polymerase (RdRp) domain of SsEV3 (Fig. 8a). The full-length sequence of *sscle_03*g029490 gene is 454 bp and comprises an ORF with two exons, encoding a hypothetical protein of 67 amino acids (Fig. S6b). Orthologous genes were found to be widely distributed among ascomycetes, all encoding hypothetical proteins with unknown functions (Fig. S6b). Based on these features, the gene was designated as *Sshp1. Sshp1* was upregulated upon SsEV3 infection. Three deletion mutants of *Sshp1* were obtained using a split-marker approach and confirmed by serial PCR (Fig. S7c). The deletion mutants showed no difference in growth rate, colony morphology, or acid accumulation compared to strain SCH941A1 (Fig. 8b and c; Fig. S10). However, the lesion diameters on the detached rapeseed leaves of the deletion mutants were significantly reduced, and infection cushion formation was impaired at 12 hpi (Fig. 8b and d).

Reactive oxygen species (ROS) play a crucial role in plant defense against pathogen invasion (31). To evaluate the role of the gene *Sshp1* in the resistance of *S. sclerotiorum* to oxidative stress triggered by plant ROS bursts, all transformants were inoculated onto PDA supplemented with varying concentrations of H_₂_O_₂_. The ∆*Sshp1* deletion strain exhibited increased sensitivity to exogenous H_2_O_2_, with a greater inhibition of growth rate than strain SCH941A1 on PDA containing 20 mM H_2_O_2_ (Fig. S11). To assess the effects of *Sshp1* on SsEV3 accumulation, SsEV3 was successfully transferred into the deletion mutants via hyphal fusion and confirmed through RT-PCR (Fig. 8e and f). This new SsEV3-infected strain, ∆Sshp1V, exhibited slower growth with more deformed colony morphology (Fig. 8g), compared to strain SCH941A1V. qRT-PCR analysis revealed that SsEV3 accumulation was 3.0- to 3.9-fold higher in ∆Sshp1V compared to SCH941A1V, indicating enhanced SsEV3 replication in the absence of *Sshp1* (Fig. 8h). Additionally, the expression levels of *Ssdcl1*, *Ssdcl2*, *Ssago1*, and *Ssago2* were determined using qRT-PCR to understand the molecular mechanisms of the increased SsEV3 accumulation. We found that the *Ssdcl1*, *Ssdcl2*, *Ssago1*, and *Ssago2* were downregulated in ∆Sshp1V (Fig. S12). These findings suggest that *Sshp1* plays a significant role in response to SsEV3 infection and could be related to typical RNAi processes in *S. sclerotiorum*.

## DISCUSSION

The interaction between mycoviruses and their fungal hosts provides a valuable system for studying fungal pathogenicity and antiviral mechanisms, as demonstrated by the *C. parasitica* and hypovirus interactions (3). Although significant progress has been made in understanding the interactions between CHV1 and *C. parasitica* (3, 32), research on the interactions between mycoviruses and *S. sclerotiorum* is limited, and endornavirus and fungi interaction systems have not been established so far. In this study, we explored the interaction between the hypovirulence-related endornavirus SsEV3 and *S. sclerotiorum*. Our findings demonstrate that SsEV3 infection upregulates RNAi-related genes and downregulates genes associated with virulence factors. Notably, we identified two SsEV3-regulated genes with novel biological functions in infection cushion formation and antiviral response in *S. sclerotiorum*.

SsEV3 infection downregulates genes related to virulence factors in *S. sclerotiorum*, including a gene that encodes a protein that directly interacts with SsEV3 and affects infection cushion formation. Hypovirulence-associated mycoviruses often reduce the virulence of phytopathogenic fungi by suppressing the expression of virulence-related genes. In *S. sclerotiorum* and *Bipolaris maydiss*, virulence-associated genes, and cell-wall degradative enzyme-related genes are downregulated following infection with SsHADV1 and Bipolaris maydis partitivirus 36 (21, 22, 33, 34). Similarly, Botrytis cinerea hypovirus 1 causes hypovirulence by suppressing infection cushion formation in *Botrytis cinerea* (35), a phenomenon also observed in *Rhizoctonia solani* and *Beauveria bassiana* following viral infections (36, 37). In the present study, SsEV3 infection resulted in virulence debilitation and impaired infection cushions formation in the strain SCH941A1V, which corresponded to the downregulation of genes related to virulence factors, including infection structures, cell-wall degradative enzymes, and secretory proteins. This suggested that SsEV3 weakened the virulence of *S. sclerotiorum* by affecting the expression of these genes. Additionally, we found that gene *Sssnf1* interacts with the Mtr domain of SsEV3, and the gene plays a vital role in infection cushion formation and pathogenicity. Our results provide new insights into the pathogenesis of *S. sclerotiorum*, highlighting how the investigation of unique phenotypes caused by hypovirulence-associated mycoviruses helps elucidate fungal pathogenesis.

SsEV3 infection triggered the RNAi antiviral processes in *S. sclerotiorum*. In *C. parasitica*, key RNAi-related genes, including *dcl2* and *ago2*, are upregulated in response to CHV1 infection and play key roles in its antiviral response (11, 38). Recent research demonstrates the presence of a Dicer-alone antiviral defense mechanism against RNA viruses in *C. parasitica*, with different RNA viruses exhibiting varying degrees of susceptibility to this defense mechanism (32). Similarly, in *S. sclerotiorum*, *dcl1* and *dcl2* are essential for interactions with SsHV2-L and SsHADV1 (39), and *ago2* has been implicated in the defense against SsHV2-L (40). Our study showed the upregulation of five RNAi pathway genes upon SsEV3 infection, which is consistent with findings in *C. parasitica* (41). Deletion strain ∆Ssdcl2V showed abnormal colony morphology with a marked reduction in growth rate compared with strain SCH941A1V and with a significantly higher accumulation of SsEV3 and a reduced siRNA derived from SsEV3. Therefore, SsEV3 infection can trigger an RNAi antiviral response in *S. sclerotiorum*, further validating that the RNAi antiviral pathway is a conserved mechanism in fungi, regardless of the type of mycovirus.

*Sshp1* encodes a hypothetical protein that plays an important role in defense against SsEV3 infection. RNAi is recognized as a significant antiviral mechanism; however, its transcriptional regulatory mechanisms remain largely unclear. In *C. parasitica*, the Spt–Ada–Gcn5 acetyltransferase (SAGA) complex regulates the expression of *dcl2* and induces the expression of a subset of genes. Here, *dcl2* has dual functions: it acts as an antiviral agent by processing virus-derived dsRNA into siRNAs and participates in transcriptional induction mediated through the SAGA complex (42, 43). CpGap1 has been identified as an antiviral factor in response to CHV1 infection (44). Additionally, genes that influence viral replication or accumulation have been identified in other fungi. For instance, *CfSnc1* and *CfKOB1* affect RNA accumulation of Colletotrichum alienum partitivirus 1 in *Colletotrichum fructicola* (45, 46), whereas *HEX1* and *FgHal2* are essential for RNA accumulation of Fusarium graminearum virus 1 in *Fusarium graminearum* (47, 48). However, the direct interactions between fungal genes and mycoviruses have not yet been reported. In this study, *Sshp1* was found to interact directly with the RdRp domain of SsEV3. The accumulation of SsEV3 was significantly elevated in the *Sshp1* deletion mutants. These results suggest that *Sshp1* is involved in viral RNA replication and has a crucial antiviral role in *S. sclerotiorum*. Furthermore, the expression of *dcl2* was downregulated in the deletion mutants (Fig. S12), indicating that the deletion of *Sshp1* inhibited *Ssdcl2* expression. This phenomenon resembles the regulation of *dcl2* by the SAGA complex in *C. parasitica*. However, whether *Sshp1* regulates the expression of *Ssdcl2* in a manner similar to the SAGA complex requires further investigation.

In conclusion, this study established a model to investigate the interaction between SsEV3 and *S. sclerotiorum*, serving as an example of an endornavirus-fungus interaction system. These findings highlight the multifarious effects of SsEV3 on *S. sclerotiorum* and provide new insights into the interactions between endornaviruses and their fungal hosts.

## MATERIALS AND METHODS

### Fungal strains and culture conditions

*S. sclerotiorum* strain SCH941A1V, infected by SsEV3, exhibits typical hypovirulent phenotypes (26), while the mycovirus-free strain SCH941A1 shows strong virulent traits. All strains were cultured on PDA at 20°C and maintained on PDA slants at 4°C.

### Phenotypic measurement of *S. sclerotiorum* strains

#### Colony morphology and virulence assay

Colony morphology and virulence assays of *S. sclerotiorum* strains were conducted as previously described (49). Organic acid production was measured based on the color change of pH-indicator solid media, that is, PDA supplemented with 50 mg/L bromophenol blue. The medium turns yellow as the pH decreases below 3.0, and the intensity of this color change indicates the level of organic acid accumulation; conversely, when the pH increases above 4.6, the PDA turns blue (50). Based on pH change profiles, the growth of *S. sclerotiorum* can be divided into two stages: the first stage is associated with a decrease in pH, while the second stage is associated with an increase in pH (51). Mycelial plugs (6 mm in diameter) were placed on PDA containing bromophenol blue (15 mL per plate). Images were taken on 3, 10, and 20 days after inoculation. Each strain was tested with a minimum of three replicates. Significant differences were analyzed using the least significant difference test, with a *P* < 0.05 considered statistically significant.

#### Microscopic observations of the infection cushion

Mycelial agar plugs, 6 mm in diameter, from the edge of the colony were inoculated onto detached rapeseed leaves or glass slides. These leaves or glass slides were then placed in an incubator with 100% relative humidity at 20°C. Samples were collected at 12, 20, and 36 hpi and stained with 0.05% trypan blue (20% lactic acid, 20% phenol, 40% glycerol, and 20% ddH_2_O). The samples were then decolorized using a solution of 25% acetic acid and 75% ethanol. Formation of the infection cushion was observed under an electron microscope (ECLIPSE Ci-S, Nikon, Tokyo, Japan), and images were captured. Each experiment was replicated at least three times.

#### Sclerotial morphology observation

To observe and assess the sclerotia produced by *S. sclerotiorum* strains, mycelial agar plugs (6 mm in diameter) from the edge of the colony were cultured on PDA (15 mL per plate) at 20°C for 30 days. The sclerotia were collected and dried, and their weights and numbers per plate were recorded. Each strain was cultured for 10 PDA plates.

#### Abiotic stress sensitivity assay

To assess the sensitivity of *S. sclerotiorum* to abiotic stresses, including salts, sugar alcohols, sugar, and cell wall stressors (Congo Red and SDS), mycelial agar plugs (6 mm in diameter) were cultured on PDA supplemented separately with 0.5 M salts (CaCl_2_ and MgCl_2_), 0.8 M salts (NaCl and KCl), 1 M sugar alcohol (sorbitol and mannitol), 1 M sucrose, or cell wall stressors (0.5 mg/mL Congo Red and 0.01% SDS). The colony morphology was observed and photographed at 36 hpi. The growth inhibition rate (GIR) of a substance was calculated using the formula: GIR = (*A* − *B*)/*A* × 100%, where *A* represents the growth rate of *S. sclerotiorum* on the PDA in the absence of stressors and *B* represents the growth rate of *S. sclerotiorum* on the PDA containing the respective substances.

### Transmission electron microscopy observation of hyphae

The mycelia of strains SCH941A1 and SCH941A1V were collected after growth on PDA at 20°C for 2–3 days and cut into small pieces (1 × 1 × 2 mm). The samples were fixed in 4% glutaraldehyde at 4°C for more than 6 h and sectioned according to a previously reported method (52). The ultra-structure of the hyphal cells was then observed under a 200 kV electron microscope (Talos L120C, Thermo Fisher Scientific, Waltham, MA, USA).

### Sample collection and total RNA extraction

Strains SCH941A1 and SCH941A1V were cultured on cellophane membranes overlaying PDA for 3 days. Approximately 1 g of mycelia per strain was collected and subjected to total RNA extraction using a TRIzol RNA extraction kit (TaKaRa, Dalian, China) following the manufacturer’s instructions. The extracted RNA was treated with DNase I (TaKaRa, Dalian, China) to remove contaminating genomic DNA. RNA quality was assessed by measuring the A260/A280 ratio using a NanoDrop 2000 spectrophotometer (Thermo Fisher Scientific, Waltham, MA, USA) and presence was confirmed using 1% agarose gel electrophoresis.

### RNA sequencing and analysis

Sequencing was performed on an Illumina HiSeq 4000 (Novogene, Tianjin, China). Low-quality reads, such as those containing adapters and those with a high content of unknown bases (N) were filtered to obtain clean reads (BioProject ID: PRJNA1135930). These clean reads were mapped to the genome of *S. sclerotiorum* using HISAT2 (53), and aligned reads were extracted using SAMtools (v1.9) (54). StringTie (v1.3.4) (55) was employed to assemble the GTF file for each sample, followed by the extraction of gene expression in ballgown format. Fragments per kilobase of exon per million and transcripts per kilobase of exon model per million were calculated for all genes in each sample. Differential gene expression was analyzed using the DESeq2 package in R-Studio (56). DEGs were identified based on absolute values of log 2-fold change (log FC) >1 and *P*-value adjusted (*P*-adjusted) false discovery rate <0.05.

Functional annotation of genes was conducted using Blast2GO (57) and the KEGG database (http://www.genome.jp/kegg/, accessed on 3 December 2021). GO and KEGG enrichment analyses were performed using the TBtools software (58). Additionally, the Pearson correlation coefficient, PCA, and volcano plots were generated for the expression data using R-studio.

### *S. sclerotiorum* genes knockout

The split-marker approach, as previously described, was used to generate gene knockout strains (59). The strategies for disrupting *sscle_01*g011030 and *sscle_03*g029490 are illustrated in Fig. S13, and the primers used to construct the plasmids are listed in Table S3. The upstream and downstream flanking sequences were amplified with primers 03g-5'F/HYR or 01g-5'F/HYR and YGF/03g-3'R or YGF/01g-3'R, respectively. Purified flanking sequences were concurrently transformed into protoplasts of strain SCH941A1 as described by Rollins (60). The hygromycin-resistant transformants were transferred onto fresh PDA containing 100 µg/mL hygromycin B. After three serial transfers, DNA was extracted from these strains and used as a template for PCR amplification. The primers TtrpCF/01g-3'DR and 01g-5'UF/PtrpCR were used to screen *sscle_01*g011030 gene knockout strains. Primers TtrpCF/03g-3'DR and 03g-5'UF/PtrpCR were used to screen *sscle_03*g029490 gene knockout strains (Table S3). Transformants were purified by the combination of hyphal tip isolation and protoplast generation, followed by selection using hygromycin (100 µg/mL).

### Horizontal transmission of SsEV3 and quantitative real-time PCR analysis

Dual-culture experiments were conducted to transfer SsEV3 to gene knockout strains. Strain SCH941A1V served as the donor strain, while gene deletion mutants ∆*Ssdcl2*, ∆*Sssnf1*, ∆*Sshp1-1*, ∆*Sshp1-3*, and ∆*Sshp1-4* labeled with the hygromycin B resistance gene were recipient strains. The recipient and donor strains were dual-cultured on PDA (9 cm) for 5 days at 20°C, and then mycelial agar discs were obtained from the colony edge of the recipient strains and transferred to new PDA supplemented with hygromycin B for further observation. After three serial transfers, total RNA was extracted as previously described, and RT-PCR was performed to detect the presence of SsEV3. The genomic DNA was then eliminated and first-strand cDNAs were synthesized using the PrimeScript FAST RT Reagent Kit with gDNA Eraser (TaKaRa, Dalian, China) according to the manufacturer’s protocols. qRT-PCR was performed on a CFX Duet Real-time PCR System (Bio-Rad) using TB Green Premix Ex Taq II (Tli RNaseH Plus) (TaKaRa, Dalian, China). The *S. sclerotiorum* actin gene (*sscle_14*g099090) was used as the internal reference, and the relative expression levels of the target genes were determined using the 2^−∆∆Ct^ method. qRT-PCR assays were repeated at least twice, each with three biological replicates. The primers used for qRT-PCR are listed in Table S3.

### Yeast two-hybrid system

The yeast two-hybrid system was used to screen for genes that interact with SsEV3 in *S. sclerotiorum*. The proteins encoded by the SsEV3 conserved domain, including RdRp (11,427–12,554 nt), Hel (6,129–6,968 nt), and Mtr (1,131–2,204 nt), were cloned into the pGBKT7 vector and transferred to the yeast Y_2_H Gold strain for self-activation and toxicity verification of the bait protein genes (Fig. S14). The successful expression of RdRp and Mtr from SsEV3 into proteins in yeast cells was demonstrated by western blot analysis using MYC as an antibody (Fig. S15). Then, these proteins from SsEV3 were used as bait proteins and co-incubated with the yeast cDNA library derived from *S. sclerotiorum*, and the proteins of the primary screening interaction were obtained. The genes *Sssnf1* and *Sshp1* obtained from the primary screening were cloned into the pGADT7 vector (Fig. S16), and the yeast Y_2_H Gold strain was co-transformed with the pGBKT7 plasmid (BD-RdRp, BD-Hel, and BD-Mtr) (Fig. S16) and the pGADT7 plasmid to verify the interaction between the proteins (61). At the same time, the proteins encoded by the SsEV3 conserved domain used as prey protein, and the genes *Sssnf1* and *Sshp1* were cloned to the pGBKT7 plasmid were co-transformed to the yeast Y_2_H Gold strain.

## Data Availability

The clean sequence reads from the transcriptomic library are available in the NCBI Sequence Read Archive (SRA) under BioProject accession number PRJNA1135930.

## SUPPLEMENTAL MATERIAL

Supplemental figures, part 1 mbio.03365-24-s0001.pdf Fig. S1 to S8.

Supplemental figures, part 2 mbio.03365-24-s0002.pdf Fig. S9 to S16.

Supplemental tables mbio.03365-24-s0003.pdf Tables S1 to S3.

ASM does not own the copyrights to Supplemental Material that may be linked to, or accessed through, an article. The authors have granted ASM a non-exclusive, world-wide license to publish the Supplemental Material files. Please contact the corresponding author directly for reuse.
