# Supplementary material for: Exploring the interaction between endornavirus and Sclerotinia sclerotiorum: mechanisms of phytopathogenic fungal virulence and antivirus
Source: mBio. 2025 Feb 19;16(3):e03365-24. doi: 10.1128/mbio.03365-24 (PMC11898685; doi:10.1128/mbio.03365-24)
Supplement: Supplemental figures, part 1 — Fig. S1 to S8. [file mbio.03365-24-s0001.pdf]

Figure S1

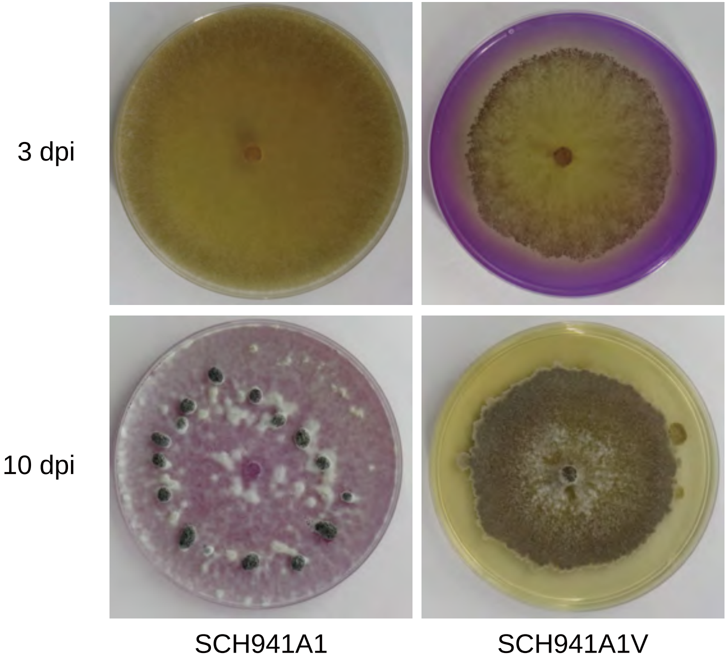

Fig. S1 Acid production of SsEV3-infected strain SCH941A1V. Infected and non-infected strains were cultured on PDA amended with bromophenol blue (50 mg/L). The photos were taken at 3 and 10 dpi.

Figure S2

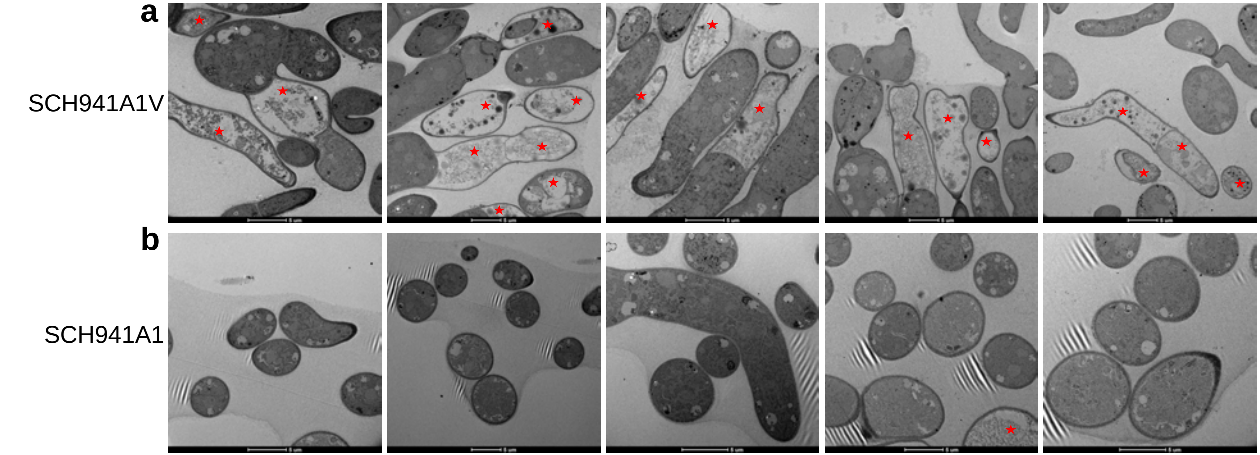

Fig. S2 Cellular ultrastructure of SsEV3-infected strain SCH941A1V. (a) Cellular ultrastructure of strain SCH941A1V as observed in the five microscopic fields. Asterisks represent vacuolated cells. (b) Cellular ultrastructure of strain SCH941A1 as observed in the five microscopic fields. Asterisks represent vacuolated cells.

Figure S3

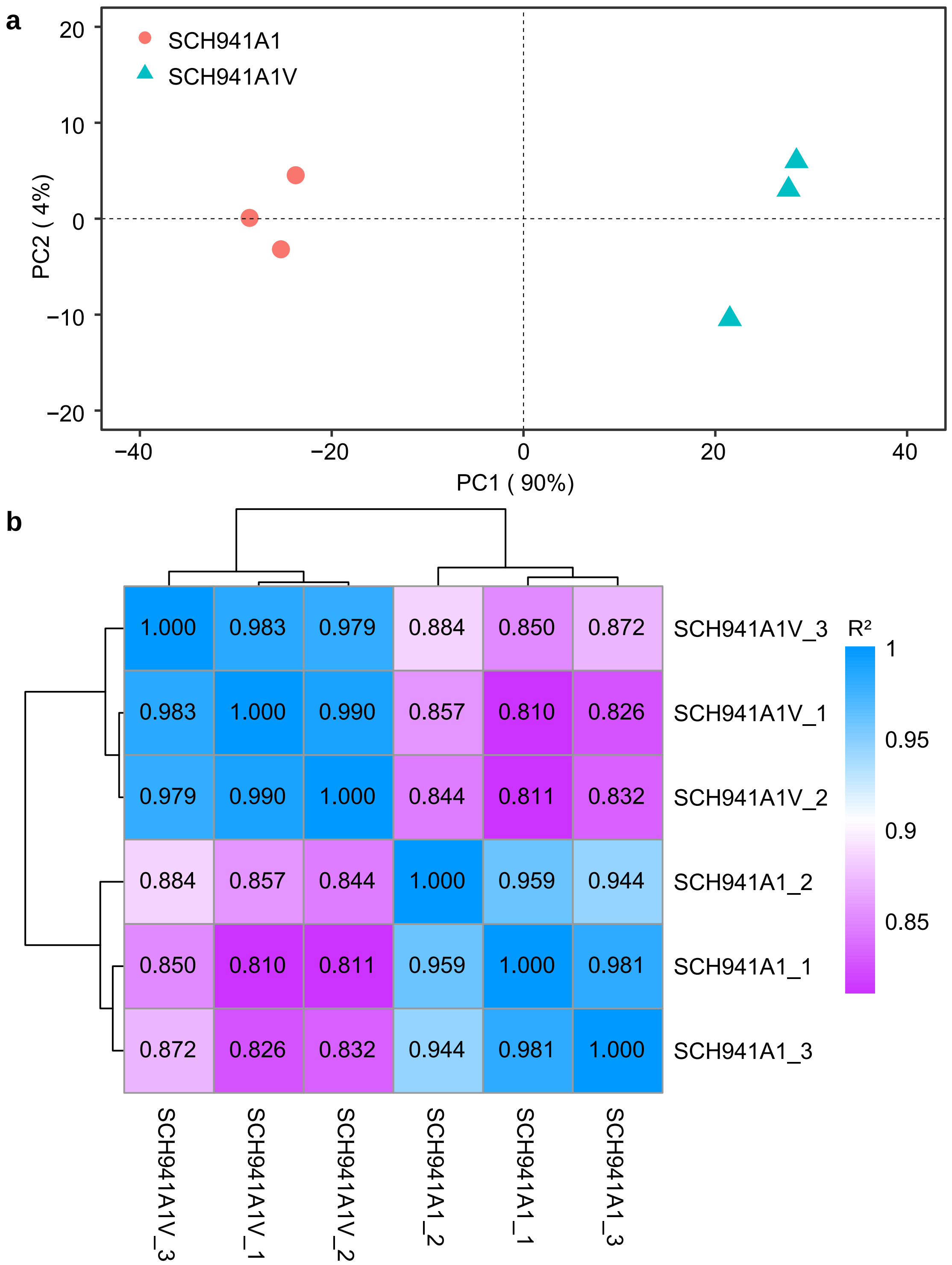

Fig. S3 Correlation analysis of gene expression in sequencing samples. (a) Principal component analysis (PCA) was performed on read counts of all sampled genes. The abscissa and ordinate respectively represent the first and second principal components. The value in the brackets of the axis label represents the percentage of the overall variance explained by the principal components. (b) Heatmap of the square value of the Pearson correlation coefficient between the sequenced samples. The squared value of the correlation coefficient of samples within and between groups was based on the read count value of all genes examined in each sample. The higher the correlation coefficient between samples, the more similar their gene expression patterns.

Figure S4

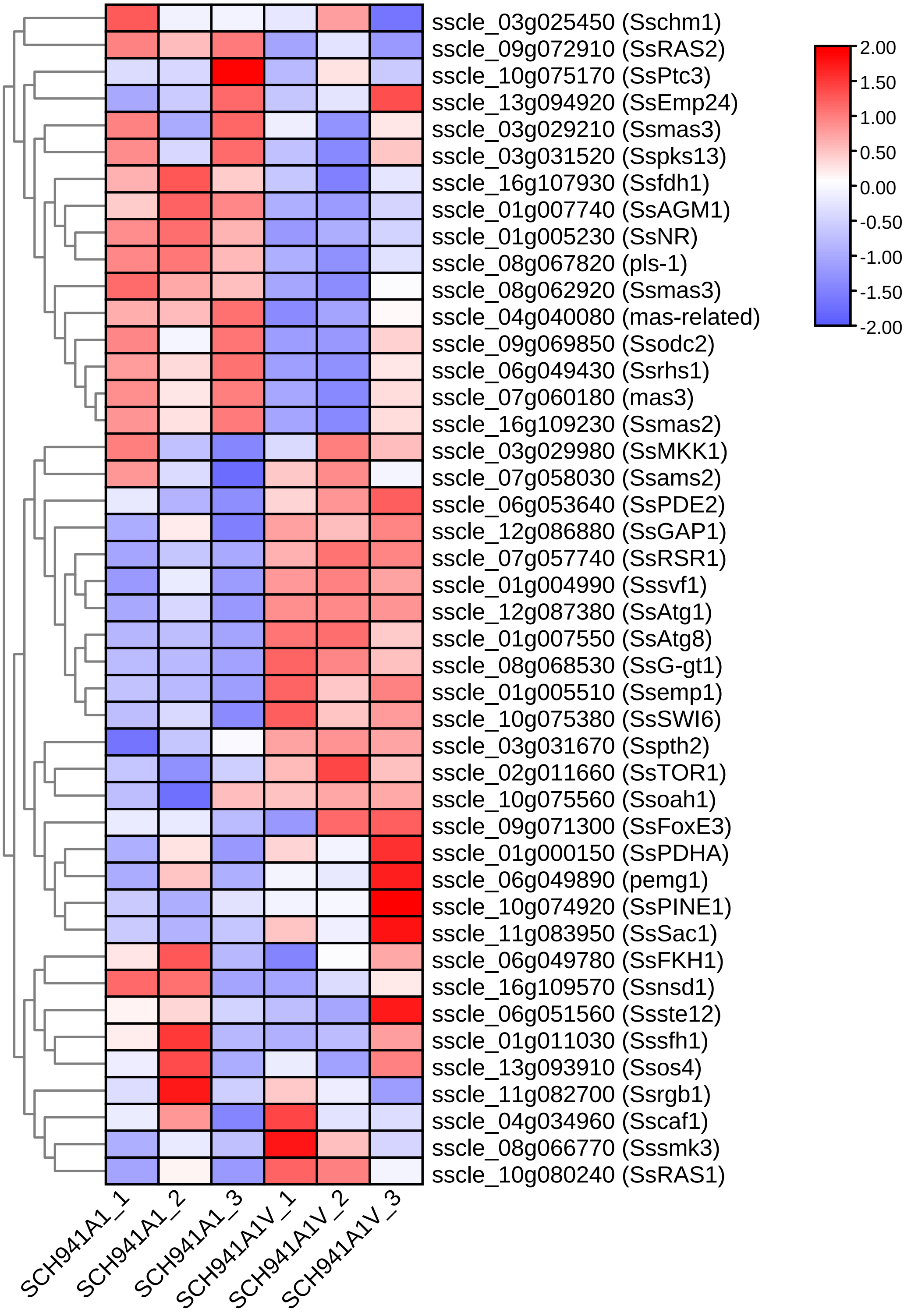

Fig. S4 Expression of genes related to infection structures in *S. sclerotiorum* and *Magnaporthe oryzae* in mycovirus-free and SsEV3-infected strains.

Figure S5

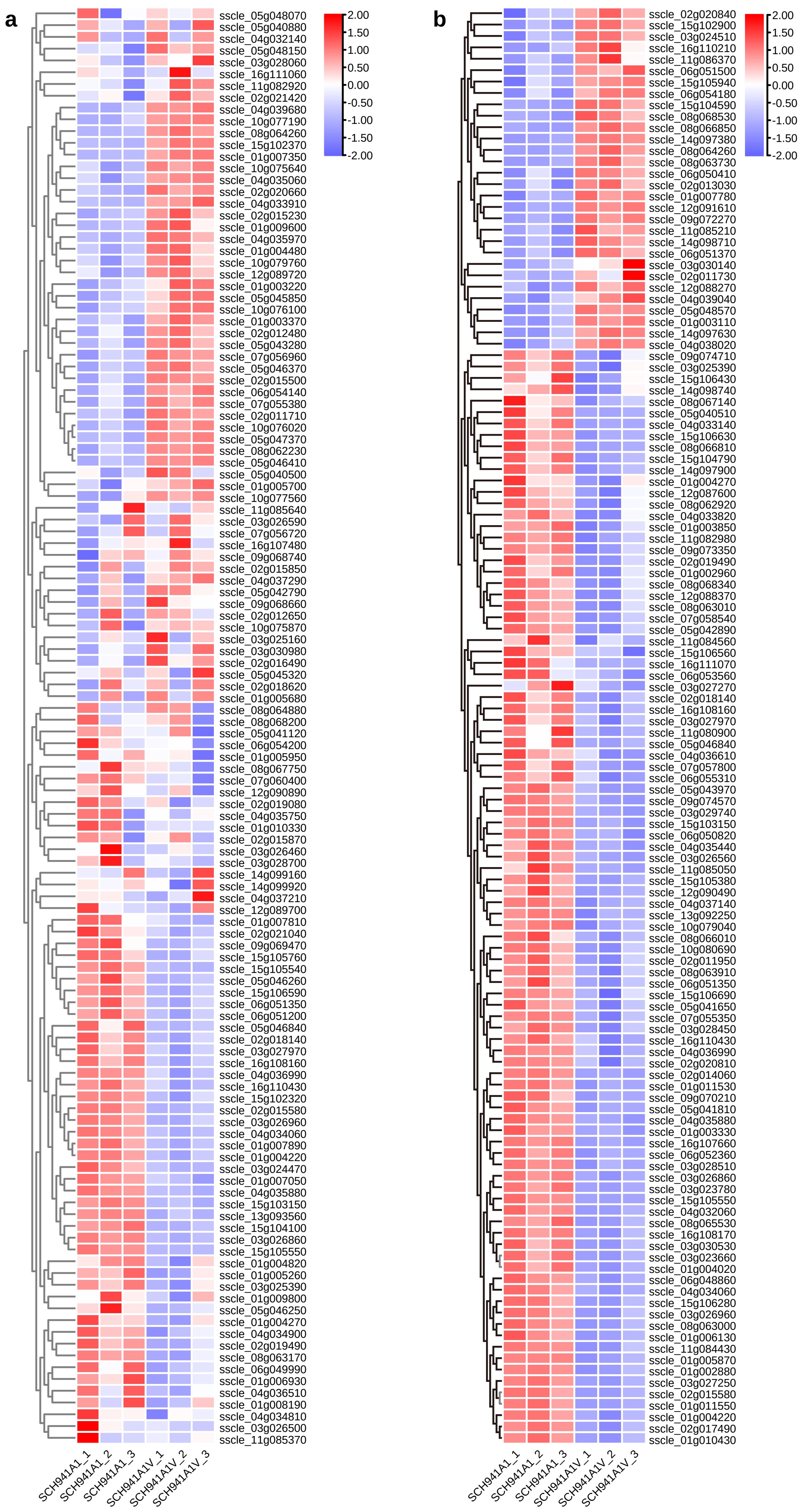

Fig. S5 Expression of DEGs related to cell wall-degrading enzymes and secreted protein in virus-free and SsEV3-infected strains. (a) The expression of DEGs related to cell wall-degrading enzymes. (b) The expression of DEGs related to secreted protein.

Figure S6

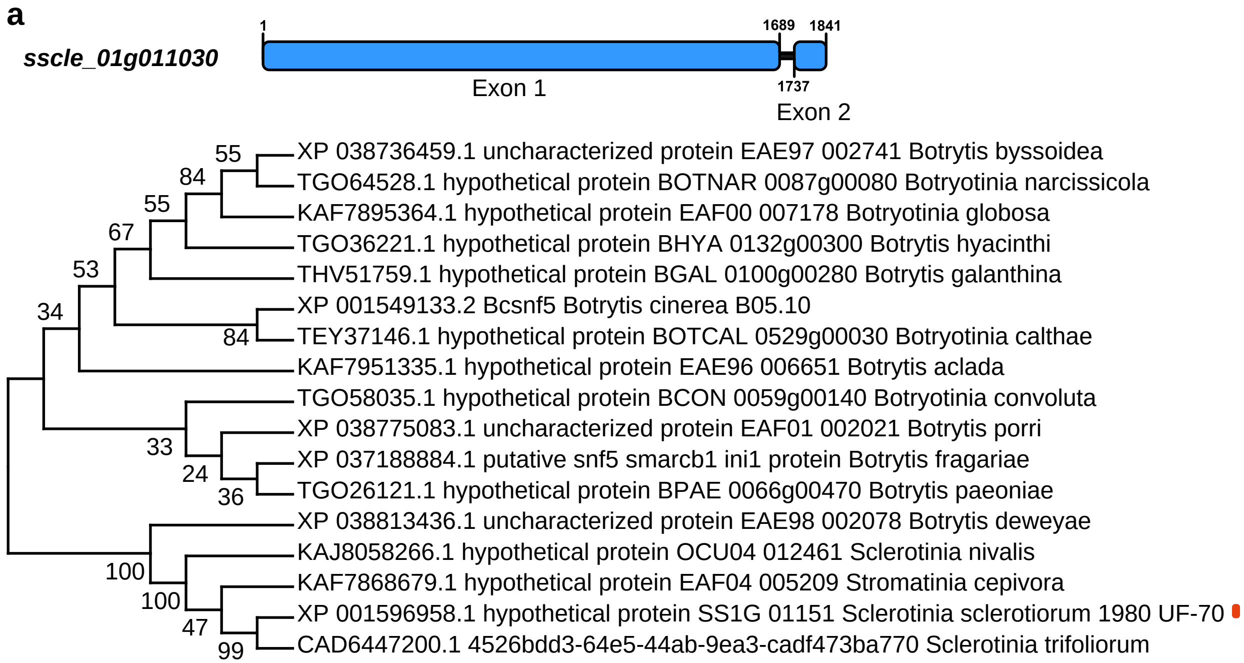

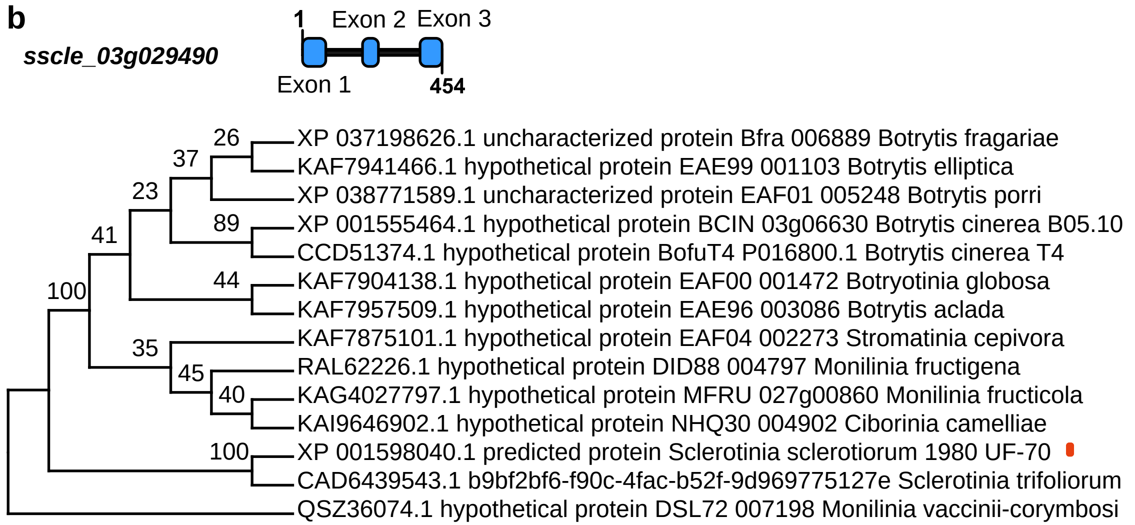

Fig. S6 Structural diagram and phylogenetic tree of the genes *sscle_01g011030* and *sscle_03g029490*.

Figure S7

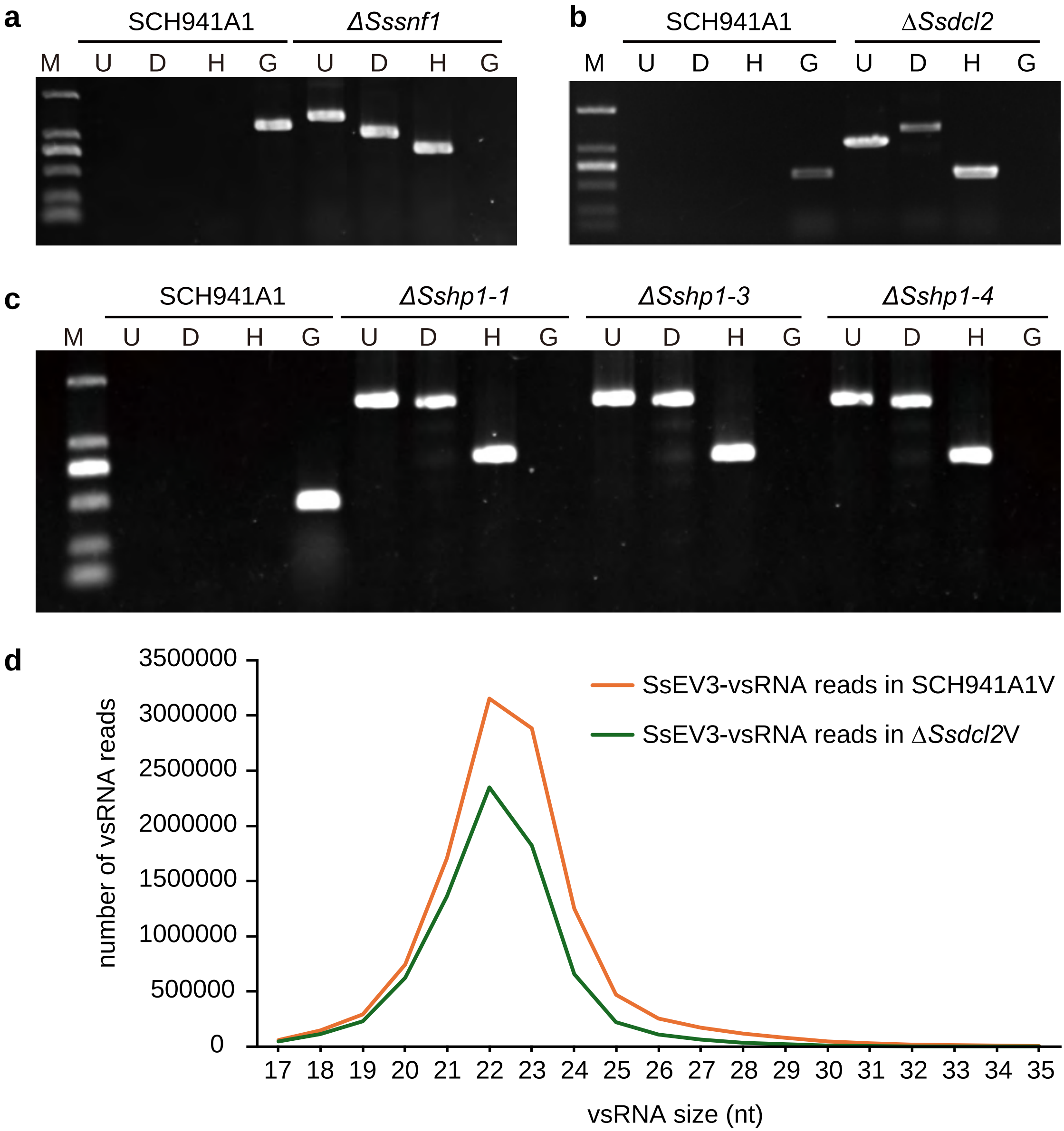

Fig. S7 Identification of the deletion mutants using PCR and the number of small RNA derived from SsEV3. (a) *Sssnf1*. (b) *Ssdcl2*. (c) *Sshp1*. (d) The number of SsEV3-derived small RNA in ΔSsdcl2V and SCH941A1V.

Figure S8

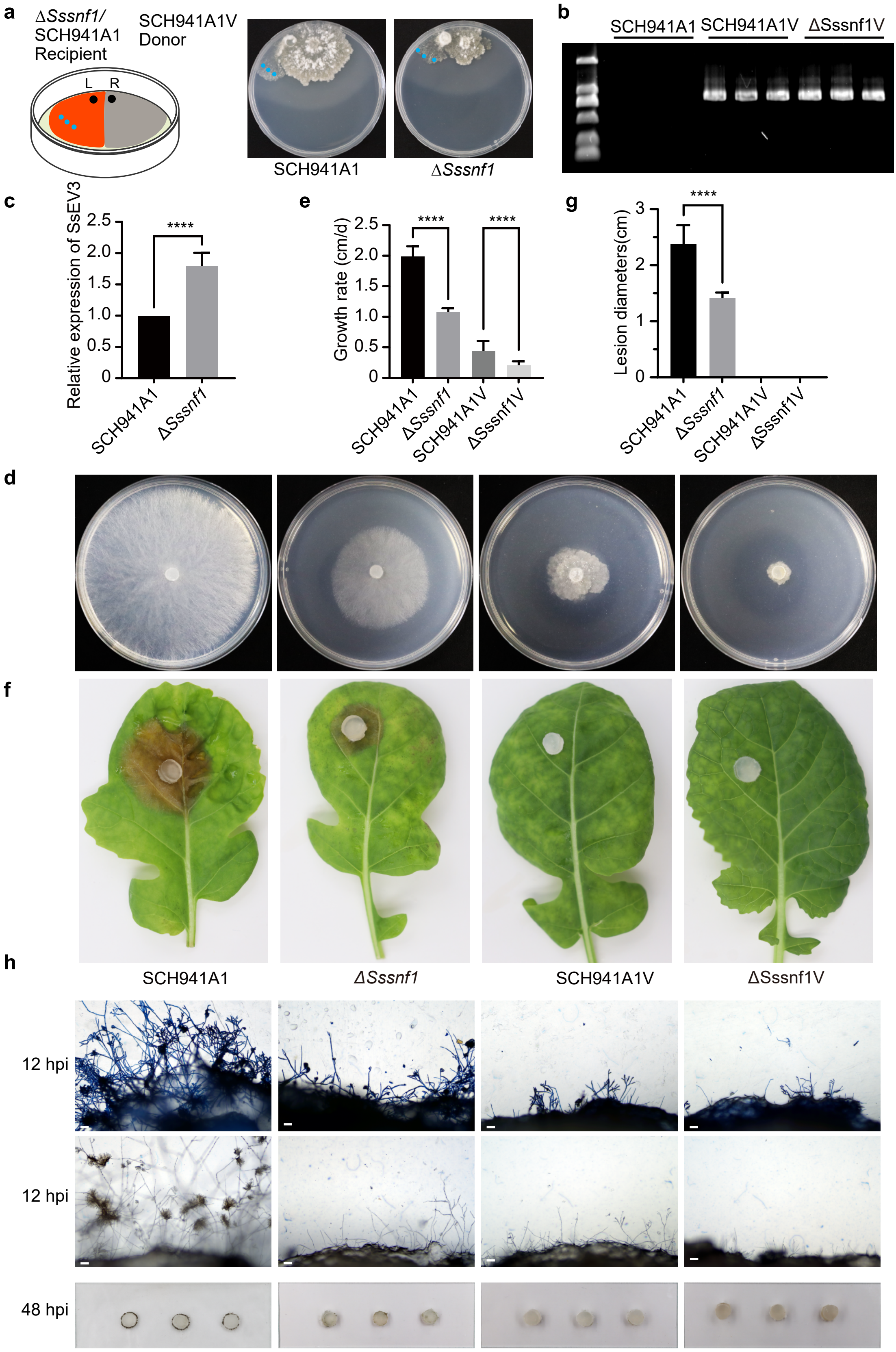

Fig. S8 Biological characteristics of the ΔSssnf1V strains. (a) Dual-culture of *ΔSssnf1* and SCH941A1V. Strain SCH941A1V on the right of plates was served as donor strain, *ΔSssnf1* was labeled with the hygromycin B resistance gene and served as recipient strain, and SCH941A1 served as recipient strain were as control. The photos were photographed at 3 d on co-culturing. (b) RT-PCR confirmation of SsEV3 in strain ΔSssnf1V and SCH941A1V. (c) Relative expression of gene from SsEV3 in Δ*Sssnf1* deletion mutants infected by SsEV3 using qRT-PCR. (d) Colony morphology of Δ*Sssnf1*, SCH941A1, ΔSssnf1V and SCH941A1V. The photos of colony morphology were photographed at 3 dpi on PDA. (e) Growth rate of Δ*Sssnf1*, SCH941A1, ΔSssnf1V and SCH941A1V at 20˚C. (f) Pathogenicity of Δ*Sssnf1*, SCH941A1, ΔSssnf1V and SCH941A1V. Photographs depicting pathogenicity and corresponding data were collected at 48 hpi on detached rapeseed leaves. (g) Lesion diameters induced by the of Δ*Sssnf1*, SCH941A1, ΔSssnf1V and SCH941A1V on detached rapeseed leaves (20˚C, 48 hpi). (h) Infection cushions of Δ*Sssnf1*, SCH941A1, ΔSssnf1V and SCH941A1V. The bar in the figures represents 50 μm.
