# Supplementary material for: Exploring the interaction between endornavirus and Sclerotinia sclerotiorum: mechanisms of phytopathogenic fungal virulence and antivirus
Source: mBio. 2025 Feb 19;16(3):e03365-24. doi: 10.1128/mbio.03365-24 (PMC11898685; doi:10.1128/mbio.03365-24)
Supplement: Supplemental figures, part 2 — Fig. S9 to S16. [file mbio.03365-24-s0002.pdf]

Figure S9

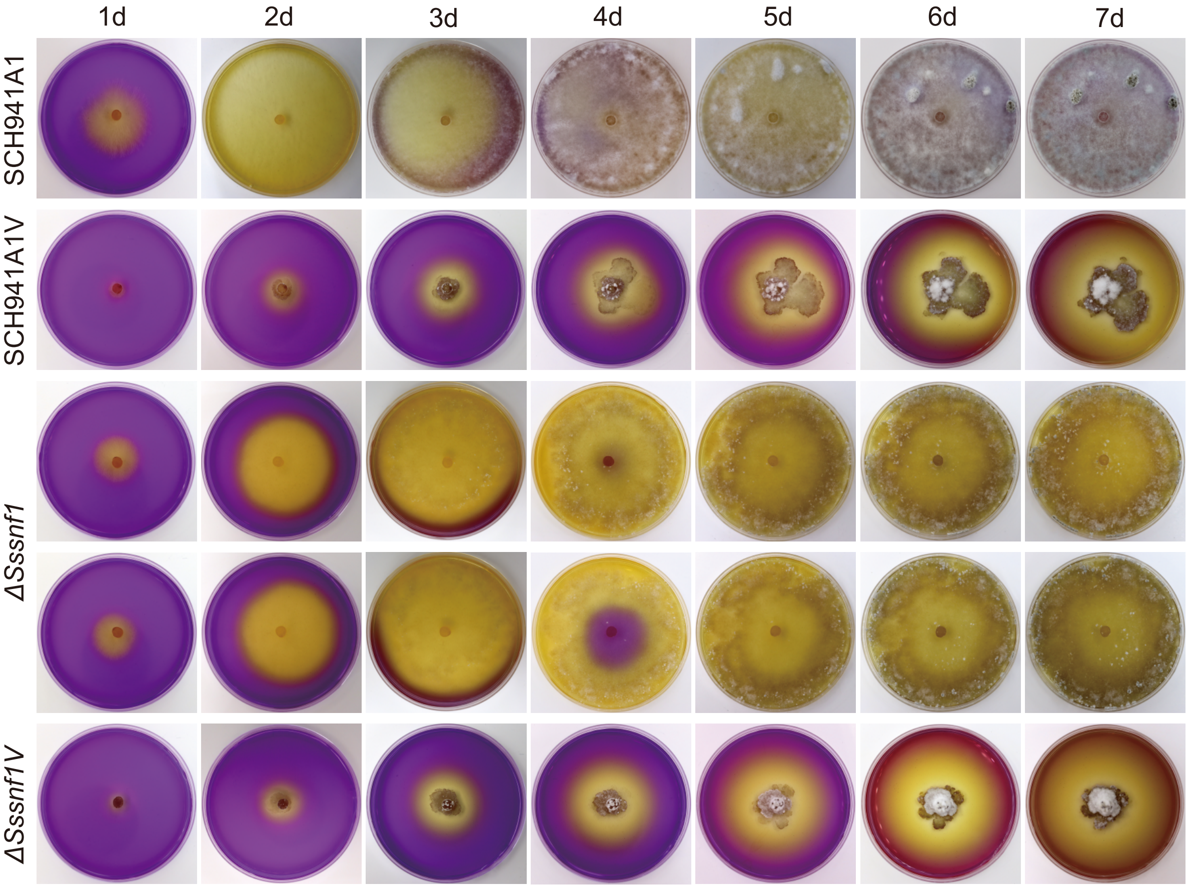

Fig. S9 Acid production of Δ*Sssnf1* deletion mutants.

Figure S10

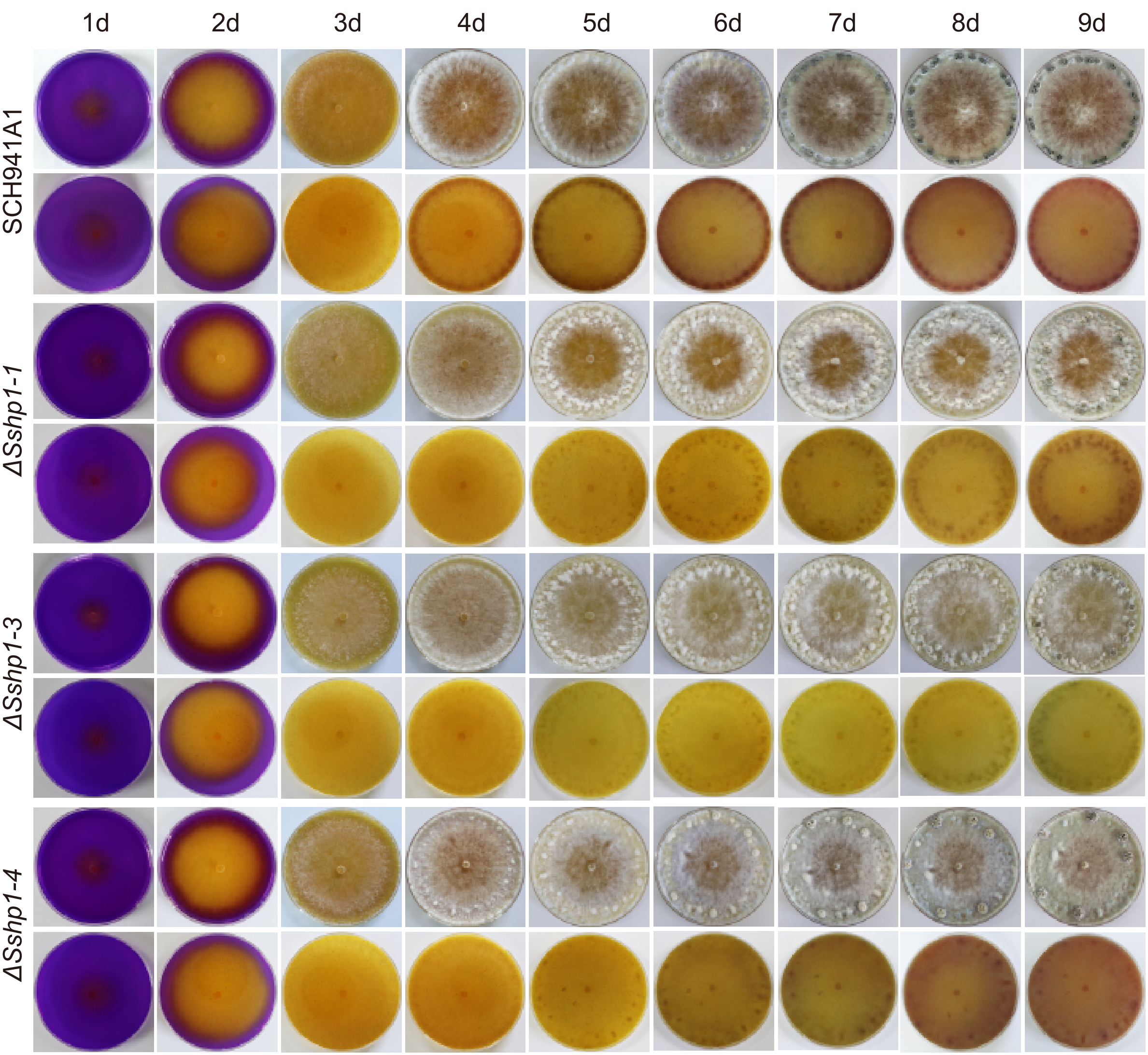

Fig. S10 Acid production of Δ*Sshp1* deletion mutants.

Figure S11

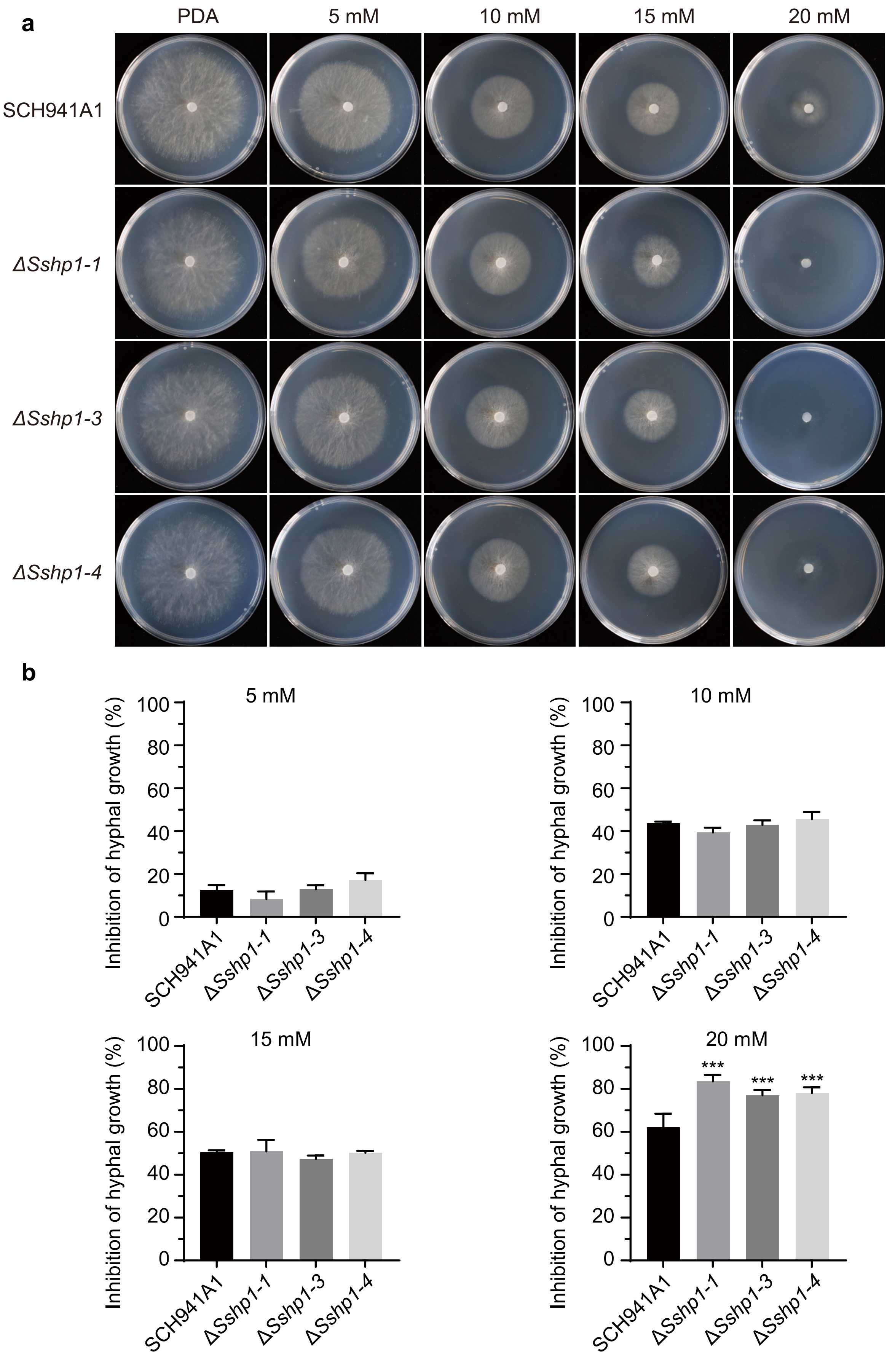

Fig. S11 Growth of the Δ*Sshp1* deletion strains on PDA containing H_2O_2. (a) Colonies of *S. sclerotiorum* strains cultivated under different H_2O_2 stresses at 20℃ for 2 d. (b) Relative growth inhibition of fungal colonies in response to various H_2O_2 stresses.

Figure S12

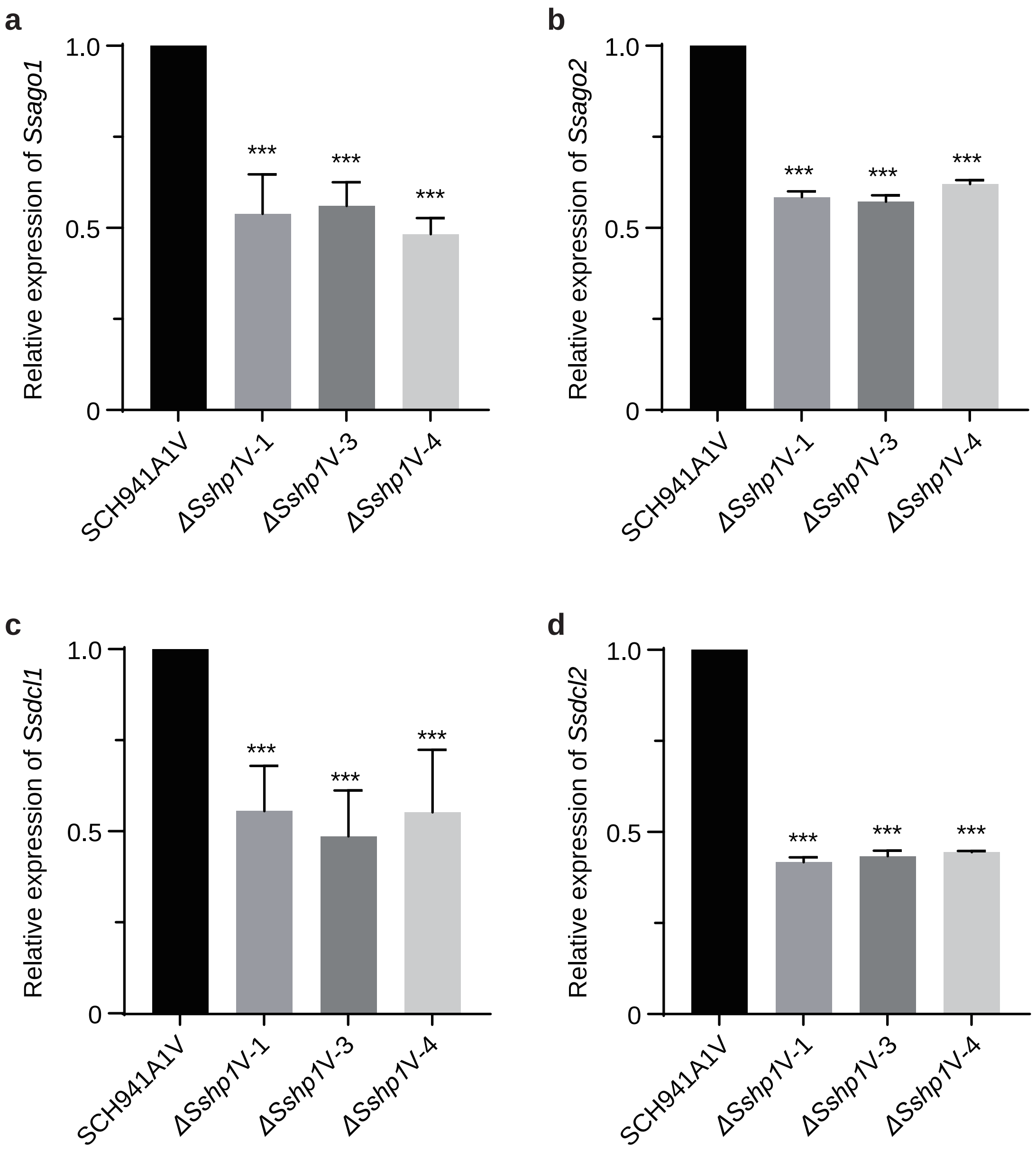

Fig. S12 Relative expression of *Ssdcl1, Ssdcl2, Ssago1*, and *Ssago2* in Δ*Sshp1* deletion mutants infected by SsEV3 and SCH941A1V using qRT-PCR.

Figure S13

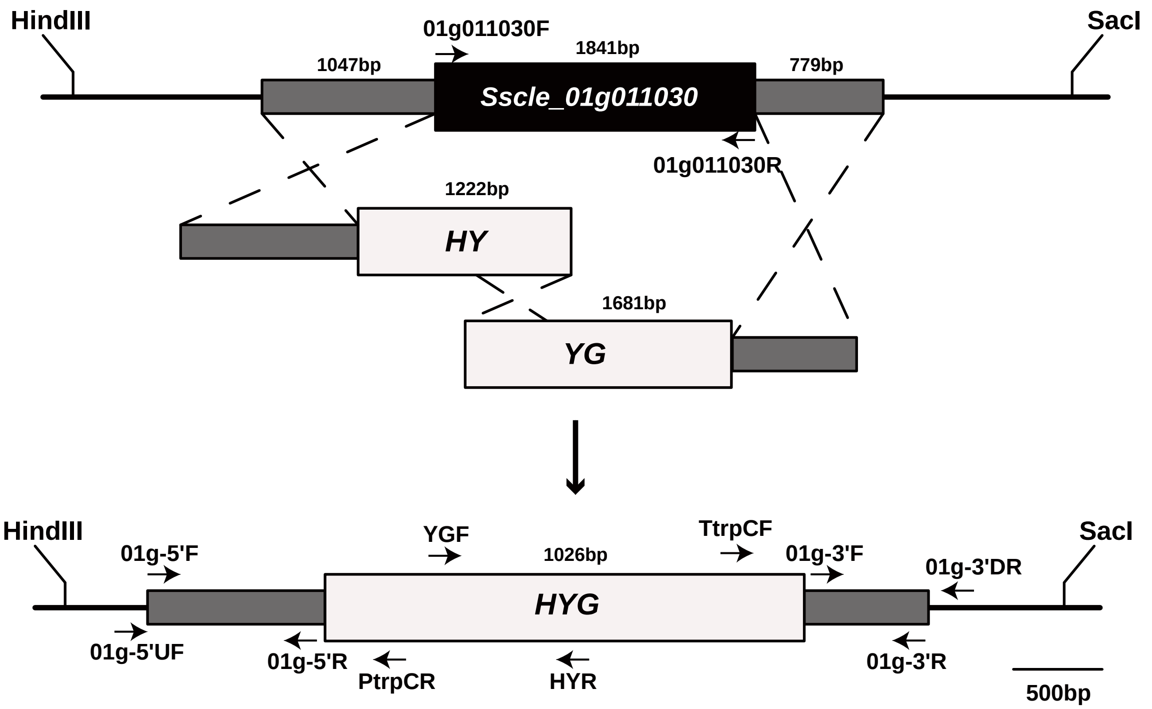

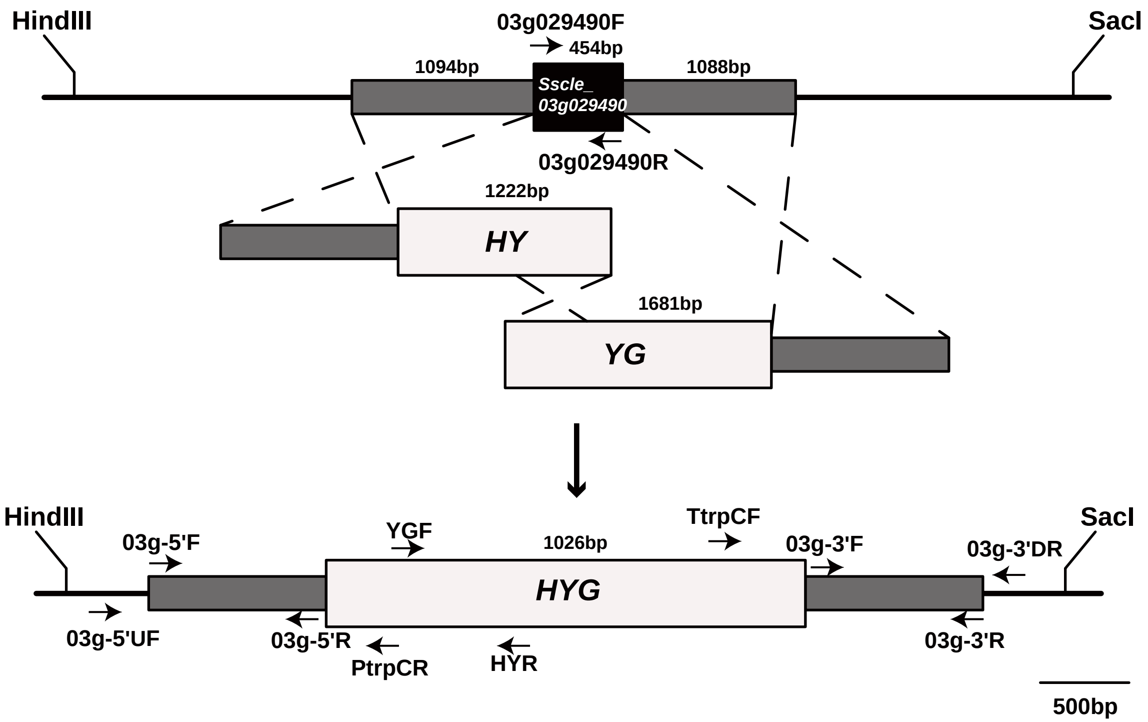

Fig. S13 Schematic diagram of the gene knockout process using split-marker approach. (a) *Sssnf1*. (b) *Sshp1*.

Figure S14

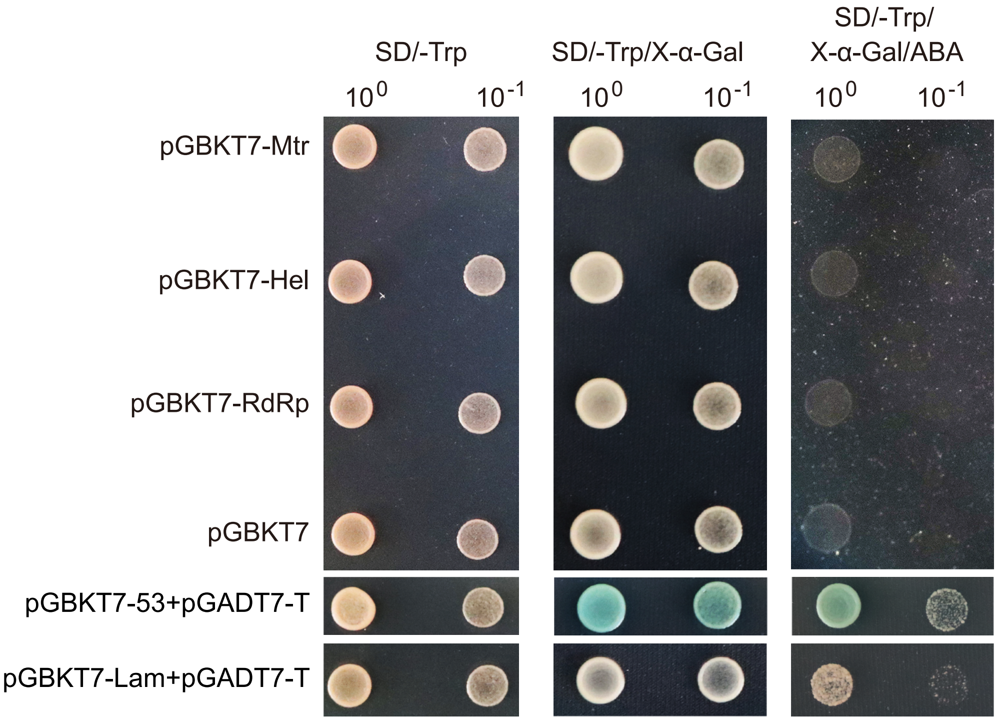

Fig. S14 Self-activation detection of pGBKT7-Mtr, pGBKT7-Hel and pGBKT7-RdRp. Positive controls: pGBKT7-53 and pGADT7-T; negative control: pGBKT7-Lam and pGADT7-T. SD/-Trp: SD/-Trp medium, SD/-Trp/X-α-gal: SD/-Trp medium containing 40 μg/mL X-α-gal; SD/-Trp/X-α-gal/ABA: SD/-Trp medium containing 200 ng/mL ABA and 40 μg/mL X-α-gal.

Figure S15

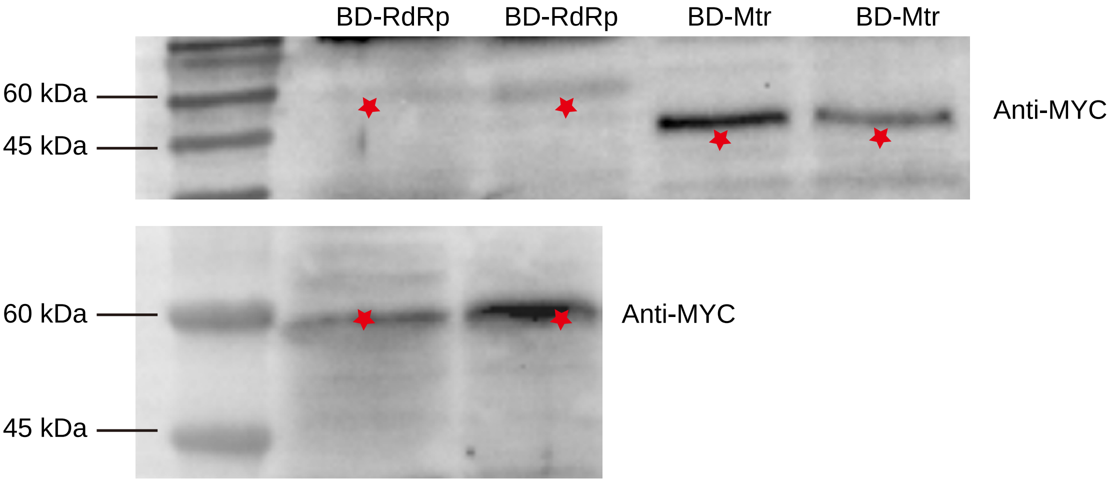

Fig. S15 Immunoblotting analysis of proteins expressed in yeast cells. The proteins from the yeast strain successfully transformed with BD-RdRP and BD-Mtr plasmids were extracted and analyzed via western blotting using a Myc antibody.

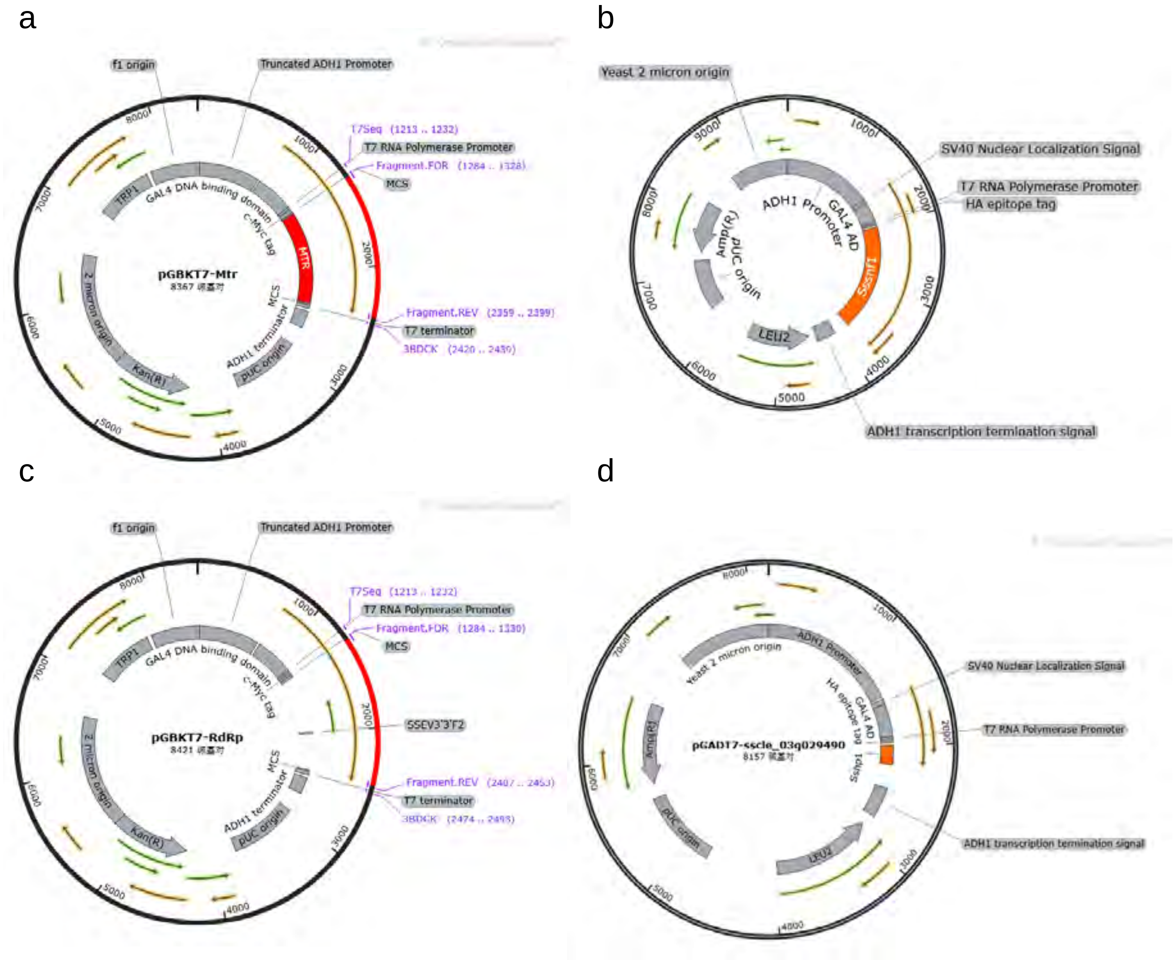

Fig. S16 Vector diagrams of pGBKT7-Mtr, AD-sscle_01g011030, pGBKT7-RdRp and AD-sscle_03g029490 used in the Yeast two hybrid. (a) pGBKT7-Mtr; (b) AD-sscle_01g011030; (c) pGBKT7-RdRp; (d) AD-sscle_03g029490.
