# Supplementary material for: Exploring the interaction between endornavirus and Sclerotinia sclerotiorum: mechanisms of phytopathogenic fungal virulence and antivirus
Source: mBio. 2025 Feb 19;16(3):e03365-24. doi: 10.1128/mbio.03365-24 (PMC11898685; doi:10.1128/mbio.03365-24)
Supplement: Supplemental tables — Tables S1 to S3. [file mbio.03365-24-s0003.pdf]

Table S1 Total number of cells and vacuolated cells in the virus-free strain SCH941A1 and SsEV3-infected strain SCH941A1V, observed using transmission electron microscopy

SCH941A1			SCH941A1V		
The number of abnormal cells	The number of cells	The proportion of abnormal cells	The number of abnormal cells	The number of cells	The proportion of abnormal cells
0	7	0.00%	3	14	21.43%
0	9	0.00%	7	15	46.67%
1	8	12.50%	3	11	27.27%
0	11	0.00%	4	17	23.53%
0	8	0.00%	4	16	25.00%
Average proportion of abnormal cells		2.50%	Average proportion of abnormal cells		28.78%

Table S2 Summary details of sequencing data

Strains	Clean reads	Clean reads mapped to *S. sclerotiorum* genome(%)	Avrage
SCH941A1_1	20968532	96.21%	96.14%
SCH941A1_2	21071334	95.95%	96.14%
SCH941A1_3	20933982	96.25%	96.14%
SCH941A1V_1	20071598	95.34%	95.23%
SCH941A1V_2	20953691	95.17%	95.23%
SCH941A1V_3	20336606	95.18%	95.23%

Table S3 The primers used in this study.

Primer Name	Primer Sequence(5'-3')	Application
01g011030F	GTTTACGAAGGGAAGATACTGGAC	Detection for sscle_01g01103
01g011030R	GCGGTTTCTCGCCTCATT	
01g-5'F1	acgacggccagtgccaagcttAGGATTACTGACGTACAAGGGAGA	Amplification for 5'Upstream
01g-5'F2	AGGATTACTGACGTACAAGGGAGA	Amplification for 5'fusion fragment
01g-5'R	tcaatatcatcttctgtcgacTTGGAGCGTGCGAAAGAACG	Amplification for 5'Upstream
01g-3'F	gaggtaatccttctttctagaATCAAACAACCACTTATTCGTTTATTC	Amplification for 3' Downstream
01g-3'R1	catgattacgaattcgagctcGAGGAGGCGAATAAGAAGACTAAGA	
01g-3'R2	GAGGAGGCGAATAAGAAGACTAAGA	Amplification for 3' fusion fragment
01g-5'UF	CGAAGGTTTTGTTGGCGTAT	Detection for 5'Upstream
01g-3'DR	AGAGGCAGCAAAGACTCAAGG	Detection for 3' Downstream
03g029490F	CGAATCTCAACAACCTCAAGC	Detection for sscle_03g029490
03g029490R	ATTCTTCGCAGATGAGACAGG	
03g-5'F1	acgacggccagtgccaagcttGTTATGTTCTATGAGGGAGGCCA	Amplification for 5'Upstream
03g-5'F2	GTTATGTTCTATGAGGGAGGCCA	Amplification for 5'fusion fragment
03g-5'R	tcaatatcatcttctgtcgacTGATGGTATTGTATTGTATTGATTGAGTT	Amplification for 5'Upstream
03g-3'F	gaggtaatccttctttctagaATCAAACAACCACTTATTCGTTTATTC	Amplification for 3' Downstream
03g-3'R1	catgattacgaattcgagctcAAGGAGGAGCACTTTCAGTAGAAGA	
03g-3'R2	AAGGAGGAGCACTTTCAGTAGAAGA	Amplification for 3' fusion fragment
03g-5'UF	CGGCAAGGCGTGAATAAG	Detection for 5'Upstream
03g-3'DR	GTCGTATAGCTCGGCATCAA	Detection for 3' Downstream
PtrpCR	CGCCGCCGCTACTGCTACAAGTG	Detection for 5'Upstream
TtrpCF	GCTCCGTAACACCCAATACGCCG	Detection for 3' Downstream
YGF	TCTCGGAGGGCGAAGAATCTCGTGC	Amplification for 3' fusion fragment/ Detection for HYG
HYR	GCATCATCGAAATTGCCGTCAACC	Amplification for 5'fusion fragment/ Detection for HYG

SsEV3-F	CACCAATGCTTCCGTCCTCCT	Detection for SsEV3
SsEV3-R	TCGTTAGATAGCGTGCGTCAGA	
SsActin-qF	GCTTGGAGAAGTCATACG	qRT-PCR
SsActin-qR	TGATGGAGTTGAAGGTAGT	
SsEV3qF1	TGATGATAGCCTGATACTC	qRT-PCR for SsEV3
SsEV3qR1	GACTCCTGAATCTTCTGA	
Ago1-qF	TAGTGGAGCGTGATATTAC	qRT-PCR for *Ssago1*
Ago1-qR	AATGACTTGGTAGTGAACA	
Ago2-qF	ATCTGCGACTGATGTATC	qRT-PCR for *Ssago2*
Ago2-qR	CGTAGAGAAGTGTCTGTTA	
Dcl1-qF	ATTGTAATCTCGTCGTTAG	qRT-PCR for *Ssdcl1*
Dcl1-qR	CTCTGTTCTTCGTTATGG	
Dcl2-qF	CTCTAGGTGGTAAGGTAATCG	qRT-PCR for *Ssdcl2*
Dcl2-qR	TGTTCATCAGCAGTCTCAAT	
